# A spontaneously immortalized muscle stem cell line (EfMS) from brown-marbled grouper for cell-cultured fish meat production

**DOI:** 10.1038/s42003-024-07400-1

**Published:** 2024-12-24

**Authors:** Ting Xue, Hongwei Zheng, Yaqi Zhao, Zhenxin Zhao, Jinwu Wang, Yue Zhang, Yaru Li, Song Wang, Yongliang Liu, Changhu Xue, Huarong Guo

**Affiliations:** 1 MOE Key Laboratory of Marine Genetics & Breeding and College of Marine Life Sciences, Ocean University of China, Qingdao, China; 2 College of Food Science & Engineering, Ocean University of China, Qingdao, China; 3 Qingdao Institute of Marine Bioresources for Nutrition & Health Innovation, Qingdao, China; 4 MOE Key Laboratory of Evolution & Marine Biodiversity and Institute of Evolution & Marine Biodiversity, Ocean University of China, Qingdao, China

**Keywords:** Muscle stem cells, Stem-cell biotechnology, Agriculture

## Abstract

Lacking of suitable fish muscle stem cell line has greatly hindered the fabrication of cell-cultured fish meat. Here, we established and characterized a spontaneously immortalized marine fish muscle stem cell line (EfMS) from brown-marbled grouper (*Epinephelus fuscoguttatus*), which could actively proliferate with good genetic stability and well maintain the stemness of myogenesis potential for over 50 passages. Taurine was found to be able to serve as a substitute of fish muscle extract in maintaining stemness. The EfMS cells could be efficiently induced to myogenic differentiation or adipogenic trans-differentiation in both 2D and 3D culture systems. Using edible 3D microcarriers, we produced 0.65 g fat-free and 1.47 g fat-containing cell-cultured fish meat in 8 days. The scaffold-free cell-cultured fish meat exhibited a much higher content of flavory amino acids than natural fish. Together, EfMS cell line can serve as an ideal seed cell line for the production of cell-cultured fish meat.

## Abstract (web summary)

Establishment and characterization of a spontaneously immortalized fish muscle stem cell line (EfMS) from brown-marbled grouper with undiminished stemness and differentiation potential for the production of cell-cultured fish meat.

## Introduction

Cell-cultured meat, also known as lab-grown meat, is generated by the in vitro proliferation and myogenic differentiation of muscle stem cells^1^. This concept was initially proposed by Winston Churchill in 1932^2^, but garnered widespread attention until 2013 when the world’s first cell-cultured beef burger was created^3^, and it developed rapidly thereafter. Today, the cell-cultured meats of beef, pork and chicken have entered the commercial stage. The production process of cell-cultured meat involves the acquisition of seed cells, in vitro proliferation, myogenic and/or adipogenic differentiation, and food-grade processing^4,5^. To obtain ideal seed cells is the first and foremost step. Adult stem cells, particularly muscle stem cells and preadipocytes, are preferred due to their higher differentiation efficiency compared to embryonic and induced pluripotent stem cells^6^.

The regenerative capacity of skeletal muscle can be attributed to the presence of satellite cells, a kind of quiescent muscle stem cells. Once activated by injury, these cells will proliferate and differentiate into myoblasts, myotubes, and finally multinuclear myocytes^7,8^. The self-renewal of muscle stem cells is driven by paired box 7 (PAX7), a transcription factor playing a crucial role in myogenesis^9,10^. The myogenic differentiation factor 1 (MyoD) involves in the regulation of the myogenic differentiation of muscle stem cells instead^11^. In the satellite cells, the activation of MyoD marks the commitment to the myogenic lineage, and as differentiation progresses, PAX7 expression decreases^12^. Accumulating evidences have showed that the in vitro cultured muscle stem cells derived from mammals always lost their stemness quickly after isolation, and rarely maintain it beyond 10 passages^13^. This necessitates frequent isolation and purification of the satellite cells from the adult tissues, thus increasing the cost of large-scale cell-cultured meat production. Therefore, to establish a continuous muscle stem cell line with undiminished stemness even after a long-term in vitro cultivation is urgently needed for the cell-cultured meat industry.

In contrast, studies on cell-cultured fish meat began much later than those in mammals and birds, and was still in the laboratory stage. In 2002, National Aeronautics and Space Administration (NASA) funded research to develop cell-cultured goldfish meat for astronauts. However, this attempt was limited to the in vitro culture of muscle tissue blocks (i.e., explants) rather than single cells, thus not a true sense of cell-culture fish meat^14^. Subsequent efforts focused on establishing muscle cell lines from various fish species, including zebrafish (*Danio rerio*)^15^, goldfish (*Carassius auratus*)^16^, rainbow trout (*Oncorhynchus mykiss*)^17^, turbot (*Scophthalmus maximus*)^18^, Atlantic salmon (*Salmo salar*)^19^, Japanese flounder (*Paralichthys olivaceus*)^20^, humpback grouper (*Cromileptes altivelis*)^21^ and sea bream (*Sparus aurata*)^22^, etc. However, these cell lines’ stemness wasn’t characterized due to the lack of Pax7 expression analysis. Until 2021, Kong et al. reported a rockfish (*Sebastes schlegeli*) muscle stem cell line which can maintain its stemness for up to 10 passages^23^. In 2022, Tsuruwaka et al. attempted to produce cell-cultured fish meat using the fibroblast cells derived from the fin tissues of thread-sail filefish (*Stephanolepis cirrhifer*)^24^. Recently, Xu et al. isolated muscle stem cells from large yellow croaker (*Larimichthys crocea*) and sub-cultured them for at least 26 times, but their stemness was maintained only to passage 3 with approximately 43% of the cells were positive for Pax7. They successfully created tissue-like fish fillets by 3D-bioprinting using myogenically differentiated muscle fibers and adipocytes of the large yellow croaker^25^. While this was a significant milestone, they failed to develop a stable, immortalized fish muscle stem cell line or preadipocyte cell line. Thus, the development of spontaneously immortalized fish muscle stem cell line (or preadipocyte cell line) with undiminished myogenesis (or adipogenesis) potential is still a big challenge persisted in the production of cell-cultured fish meat.

Brown-marbled grouper (*Epinephelus fuscoguttatus*) is a warm-water marine fish species belonging to the family Serranidae, order Perciformes. It lives in the sea area from East Africa to the Phoenix Islands. Due to its merits like rapid growth, delicious meat and rich nutrition, brown-marbled grouper is highly valued for its economic importance as a food source^26^. Up to date, there is no reports on the isolation and establishment of muscle stem cell line from brown-marbled grouper.

In this study, we established and characterized a spontaneously immortalized, marine fish muscle stem cell line (EfMS) from the brown-marbled grouper with undiminished stemness even after 50 passages. Moreover, the culture conditions for active proliferation and efficient myogenic differentiation as well as adipogenic trans-differentiation of EfMS cells in both 2D and 3D culture systems were successfully developed and used to produce cell-cultured fish meat. Together, this work provides an ideal seed cell line for the production of cell-cultured fish meat.

## Results

### Establishment of spontaneously immortalized muscle stem cell line (EfMS) from brown-marbled grouper (*E. fuscoguttatus*)

**Fig. 1:**
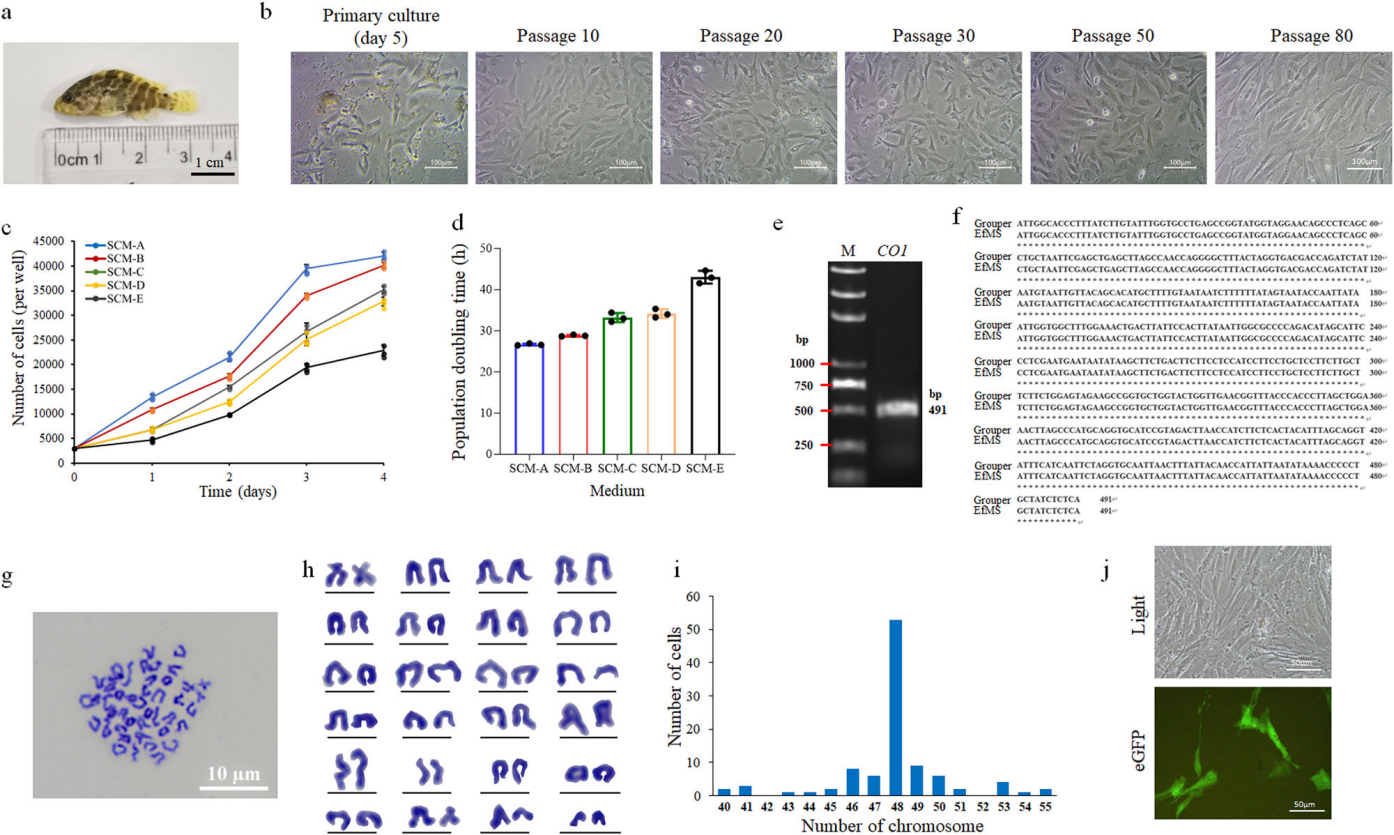
Establishment and characterization of brown-marbled grouper (*E. fuscoguttatus*) muscle stem cell line (EfMS). **a** 1-month-old brown-marbled grouper. Scale bar: 1 cm. **b** Primary culture (Day 5 after isolation) and subculture of EfMS cells at passages 10, 20, 30, 50, and 80, respectively. Scale bar: 100 μm. **c** Growth curves of EfMS cells cultured in five different media of SCM-A, B, C, D and E for 4 days in 96-well culture plates, respectively. Cell numbers were counted daily. Data shown as mean ± standard error (*n* = 3). **d** Calculated population doubling times of EfMS cells in different growth media within the first 3 days. **e** Agarose gel electrophoresis results of PCR amplification fragment (491 bp) of *CO1* gene of EfMS cells. Lane M, DNA Marker. **f** Sequence alignment of the *CO1* gene fragment of EfMS cells with the known *CO1* sequence of the brown-marbled grouper (GenBank No. NC_020046.1), showing a 100% identity. **g**–**i** Chromosomal analysis of EfMS cells. **g** Metaphase. Scale bar: 10 μm. **h** Diploid karyotype. **i** Distribution of chromosome numbers. **j** Representative bright-field and fluorescent micrographs of the EfMS cells at 48 h post transfection. Scale bar: 50 μm.

In this study, as shown in Fig.1, the muscle stem cell suspension could be successfully prepared by enzymatical digestion of the finely sliced muscle tissues of young brown-marbled grouper (Fig.1a) using dispase type II (1.25 mg mL^-1^) and collagenase D (5 mg mL^-1^), in an isolation efficiency of 4.7 × 10^4^ cells/g tissues, and formed a confluent cell monolayer within 2 weeks, with half medium change every two days. Both long spindle-like and multipolar-like cells could be found in the primary cell culture with a cell size ranging from 50 to 100 μm in length. The primarily cultured grouper muscle stem cell monolayer could be successfully sub-cultured by 0.0625% trypsin-EDTA solution at a splitting ratio of 1:2 and became a new marine fish muscle stem cell line, designated as EfMS. After that, the EfMS cells were routinely maintained by half medium change every 2 days and subculture every 4 days. After passage 20, the EfMS cells showed higher tolerance to the trypsin-EDTA solution and could be well sub-cultured by higher concentration of trypsin-EDTA (0.25%). Up to date, the EfMS cell line had been passaged for over 80 times and healthily maintained for more than 500 days, inferring that it had undergone spontaneous immortalization transformation (Fig. 1b).

It was also found that the use of a rich stem cell medium (SCM-A) containing 69% L-15, 20% FBS, 10% fish muscle extract, 20 ng mL^-1^ bFGF, 20 ng mL^-1^ EGF and 1% antibiotic mixture could well support the growth of EfMS cells and inhibit their myogenic differentiation and loss of stemness in both the primary culture and subculture. In contrast, the muscle stem cells isolated in the same way from adult brown-marbled groupers exhibited very slow growth or proliferation capacity and failed to form a confluent cell monolayer.

The effects of various concentrations of fetal bovine serum (FBS) (10% and 20%), fish muscle extract (10%), and growth factors (20 ng ml^−1^ bFGF and 20 ng ml^−1^ EGF) on the growth of EfMS cells at passage 5 was evaluated, and found that the EfMS cells could grow well in all of the tested five different media (SCM-A ~ E) (Fig. 1c, d), but showed the highest growth rate in SCM-A with a population doubling time (PDT) of ~26.67 h within the first 3 days of culture, followed by SCM-B (~28.83 h/PDT), SCM-C (~33.19 h/PDT), SCM-D (~34.19 h/PDT), and the slowest SCM-E (~43.02 h/PDT). The obtained results suggested that the addition of 20% FBS, 10% fish muscle extract and growth factors of bFGF and EGF were all necessary for the optimal proliferation of EfMS cells.

### Characterization of species origin and transfection potential of EfMS cells

To confirm the species origin, the 491 bp target fragment of the mitochondrial *CO1* gene was amplified and sequenced using gene-specific primers (Fig. 1e). Sequence analysis showed that the amplified *CO1* gene fragment from the EfMS cells at passage 20 had a 100% sequence match with the known *CO1* gene of brown-marbled grouper individual, confirming that EfMS cells were originated from brown-marbled grouper (Fig. 1f). The chromosome numbers of the EfMS cells at passage 30 ranged from 42 to 54, with a mode chromosome number of 48. Moreover, most of the chromosomes were telocentric (Fig. 1g–i).

The EfMS cells had a high transfection potential with a calculated transfection efficiencies of up to 23.6 ± 3.9% when the *eGFP* reporter plasmid of pEGFP-N1 and transfection reagent of lipofectamine 8000 was used (Fig. 1j). At 48 h post-transfection, bright green fluorescent signals of eGFP could be detected in the EfMS cells, demonstrating the suitability of EfMS cells to be used for genetic manipulation.

### Characterization of the cell lineage and stemness maintenance of EfMS cell line during sustained culture

**Fig. 2:**
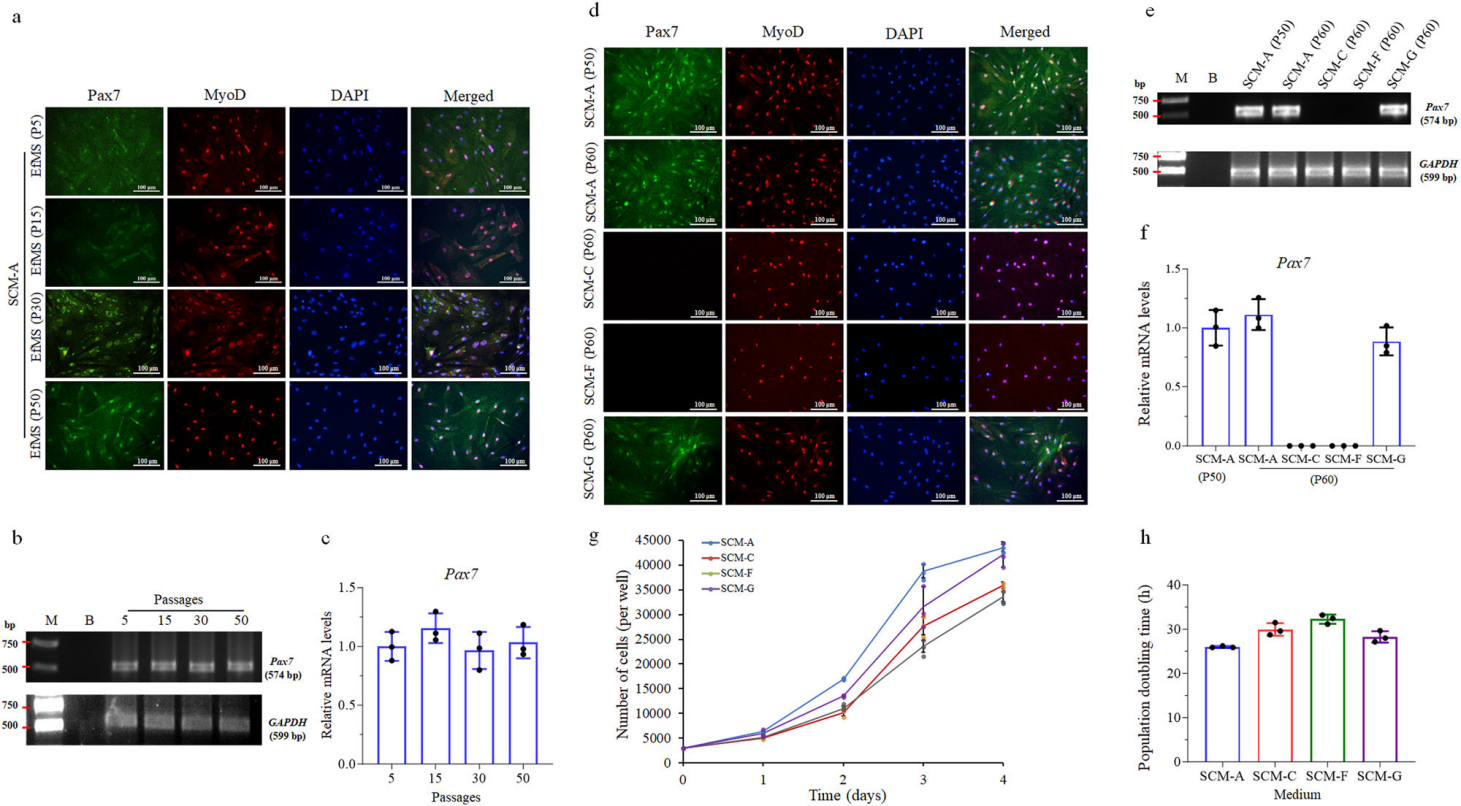
Characterization of the cell lineage and stemness maintenance of EfMS cell line. **a** Immunofluorescence staining of Pax7 (green) and MyoD (red) in the EfMS cells at passages 5, 15, 30, and 50 in SCM-A medium. Nuclei were labeled by DAPI (blue). Scale bar: 100 μm. **b** Agarose gel electrophoresis results of semi-quantitative RT-PCR amplification products of *Pax7* gene and internal reference gene of *GAPDH* from EfMS cells cultured in SCM-A at passages 5, 15, 30, and 50. M, DNA marker. B, Blank control. **c** Statistical results of gray values of each band in b, error bars indicate SD, *n* = 3. **d** Immunofluorescent staining of Pax7 (green) and MyoD (red) in EfMS cells cultured in SCM-A, SCM-C, SCM-F, and SCM-G. Nuclei were labeled by DAPI (blue). Scale bar: 100 μm. **e** Agarose gel electrophoresis results of semi-quantitative RT-PCR amplification of *Pax7* and *GAPDH* from EfMS cells cultured in SCM-A, SCM-C, SCM-F, and SCM-G. **f** Statistical results of gray values of each band in b, error bars indicate SD, *n* = 3. **g** Growth curves of EfMS cells cultured in SCM-A, SCM-C, SCM-F, and SCM-G for 4 days, respectively. Cells were counted daily. Error bars indicate SD, *n* = 3. **h** Calculated population doubling times of EfMS cells in different media within the first 3 days.

*Pax7* expression is restricted to muscle stem cells and is required for their maintenance of stemness, whereas MyoD is expressed in both activated muscle stem cells and their progeny including myoblasts, myotubes and myocytes^27^. To identify the cell lineage of EfMS cells, immunofluorescence staining for Pax7 and MyoD was performed. The results showed that Pax7 and MyoD were co-expressed in the nucleus of EfMS cells (Fig. 2a), indicating that the EfMS cells were muscle stem cells. Moreover, in the medium of SCM-A (Table 1), the stemness of EfMS cells could be maintained in vitro for at least 50 passages (Fig. 2a). The long-term stable expression of *Pax7* in the EfMS cells was also confirmed by the semi-quantitative RT-PCR results, that was, no significant difference could be observed between passages 5 and 50 (Fig. 2b, c).

**Table 1:** Formulas of the 7 kinds of fish muscle stem cell media (SCMs)

Medium	Formula^a^
SCM-A	69% L-15 + 20% FBS + 10% muscle extract + 20 ng mL^−1^ bFGF + 20 ng mL^−1^ EGF
SCM-B	69% L-15 + 20% FBS + 10% muscle extract
SCM-C	79% L-15 + 20% FBS + 20 ng mL^-1^ bFGF + 20 ng mL^−1^ EGF
SCM-D	79% L-15 + 20% FBS
SCM-E	89% L-15 + 10% FBS
SCM-F	79% L-15 + 20% FBS + 24 μg mL^−1^ Phosphoryl ethanolamine (PEA) + 20 ng mL^−1^ bFGF + 20 ng mL^−1^ EGF
SCM-G	79% L-15 + 20% FBS + 55.4 μg mL^−1^ Taurine + 20 ng mL^−1^ bFGF + 20 ng mL^−1^ EGF

*FBS* fetal bovine serum, quantified for embryonic stem cells (BI, Israel, Cat. 04-002), *bFGF* basic fibroblast growth factor (Sinobiological, China), *EGF* epidermal growth factor (Sinobiological, China).

^a^All the SCMs were also supplemented with 1% penicillin-streptomycin mixture.

To explore the role of fish muscle extract and growth factors (bFGF and EGF) in maintaining the stemness of EfMS cells, the EfMS cells at passage 5 were also cultured in the growth factors-removed SCM-B, muscle extract-removed SCM-C, both-removed and 20% FBS-containing SCM-D, both-removed and 10% FBS-containing SCM-E (Table 1), respectively, and then continuously sub-cultured for 10 times until passage 15. Both immunofluorescence staining and semi-quantitative RT-PCR analysis showed that the Pax7 expression was absent in all the EfMS cells at passage 15, but MyoD expression was maintained (Supplementary Fig. 1). This result suggested that all the tested growth media (SCM-B, C, D and E) could not maintain the stemness of the EfMS cells for a long time, in another word, both fish muscle extract and growth factors (bFGF and EGF) were essential for long-term maintenance of the stemness of EfMS cells.

### Taurine can serve as a substitute for fish muscle extract in maintaining the stemness of EfMS cells

Muscle extract played an important role in maintaining the stemness of EfMS cells. However, the use of muscle extract was labor- and time-consuming with high-cost and undefined nutrient composition, thus unsuitable for the scale-up production of cell-cultured fish meat. To screen a substitute for the muscle extract, the composition and content of free amino acids in the fish muscle extract was first analyzed. Results revealed that the top two most abundant components in the muscle extract were taurine (554 mg L^-1^) and phosphoryl ethanolamine (PEA) (240 mg L^−1^) (Supplementary Fig. 2). Both of them are absent in L-15 medium.

Notably, taurine is commonly sourced from fish and seafoods^28^, while PEA has demonstrated the ability to regulate cellular autophagy, thereby extending cellular longevity^29^. Based on this, we hypothesized that taurine and PEA may play a critical role in maintaining the stemness of marine animal muscle stem cells. To investigate this further, we supplemented the L-15 medium with taurine and PEA, respectively, at the content found in the muscle extract, to obtain the PEA-enriched medium (SCM-F) and taurine-enriched medium (SCM-G), as listed in Table 1. Then the stemness maintenance of EfMS cells at passage 50 in SCM-F and SCM-G was monitored after an additional 10 passages, and compared with the muscle extract-containing SCM-A and muscle extract-free SCM-C. The results indicated that EfMS cells could co-express Pax7 and MyoD after 10 times of subculture in SCM-A and SCM-G, but lost Pax7 expression in SCM-C and SCM-F, with MyoD expression retained (Fig. 2d). The semi-quantitative RT-PCR results also supported this finding (Fig. 2e, f). This observation indicated that taurine can effectively substitute for muscle extract in maintaining EfMS cell stemness, whereas PEA does not have this effect. Additionally, the proliferation experiments revealed that EfMS cells exhibited the highest proliferation rates in SCM-A with a population doubling time (PDT) of ~26.00 h within the first 3 days of culture, with a slightly decrease in SCM-G (~28.27 h/PDT). In contrast, the proliferation rates of cells in SCM-C (~29.94 h/PDT) and SCM-F (~32.27 h/PDT) were much lower than the former two (Fig. 2g, h). These results indicated that taurine can promote the proliferation of EfMS cells in the absence of muscle extract, while PEA does not.

### Genetic stability and safety of spontaneously immortalized EfMS cell line

To examine the impact of the spontaneous immortalization event on the genetic stability of the EfMS cell line, the transcriptional expression levels of 8 immortalization-related genes (listed in Table 2) were compared between the EfMS cells at passages 20 and 80. As shown in Supplementary Fig. 3, the expression of the three genes related to the P53 signaling pathway, *TP53*, *TP53RK* and *TP53I3*, showed no significant changes before and after the immortalization event. For the three genes of *EGFR*, *PTEN* and *MYC*, which were usually upregulated in human immortalization events, in this study, *MYC* was found to be significantly downregulated instead, whereas *EGFR* and *PTEN* showed no significant changes. For the two telomerase-related genes, *TERT* showed no significant change, whereas *DKC1* was significantly upregulated, which was in favor of the self-renewal and immortality of EfMS cells (Supplementary Fig. 3). We also found that *TERT* and *DKC1* are wildly expressed in nearly all the tested adult tissues of brown-marbled grouper at varied levels, and all of them were lower than that of EfMS cells (Supplementary Fig. 4). Taken together, it could be concluded that the spontaneous immortalization event had no adverse effects on the genetic stability of EfMS cells, let alone any signs of malignant transformation. This genetic stability underscored the potential and safety of EfMS cells as an ideal seed cell for cell-cultured fish meat production.

**Table 2:** The primers used for semi-quantitative RT-PCR

Gene	Forward (5’→3’)	Reverse (5’→3’)
Pax7	GCAGGAGTCGGACCACATTCA	TGGAGCGGTGAGATAGAGAAGTC
MHC	GCAAGAGGAATACAAGAAGGAAGG	TGGTGAAGGACAAGATGGTGAT
Myogenin	GTACGACCAATCCACCTACCA	CACTTGACGACGACACTCTG
GAPDH	CGCAAGACAGACTGAGGCTTCT	GGTGGCAGTGATGGCATGAACT
ACTB	GTTCGAGACCTTCAACACCCC	GCAGCAGTGCCCATCTCCTGCT
TP53	CCTGGTGAGGAAGGACGTTC	GGTCGGCTTGGACTTCTTCA
TP53I3	GGTGAAAGGAGACTGGAGGC	AGTTTGCCCAGCAGATCTCC
TP53RK	GGTCTCAGAGCTCCTCAGGA	CTTATCCTCTGGCAGAGCGG
MYC	TACGACTACGACTCCCTGCA	GGCGTCTCTGCAATCGGATA
PTEN	GTCGTGCGGTTTTTGGACTC	TCAGTTTGTCTTCCCGTCGG
EGFR	CTGGAGATCGTTCGAGGACG	AGAACAGGCCGTCTTGGAAG
TERT	TGGCTGAGCTGATGTGGAAG	ATCTGTCATGCCCCACACTG
DKC1	GACTCTGTGTGTCCACCTGG	TTCCCGTGTTTGTCGAGGAG
CD73	AACCCCATCCTGCTGAACAG	ATGTAGGAAGGCAGCACCAC
CD105	GTTCTCTCTCGAGATGGCCG	AAGATCCATGTGGTGCCCTG
PPARγ	GCAGCGCTAAACATCGAGTG	ATACTCTGACCCCGGGTGAA
C/EBPα	TTCTTCTTGGACTTCCCGCC	GTGAGATCGGAGACAGCGAG
LPL	CTGTGCAGAGTGTGACGTCT	GTTGCATGGCTTCCAGCAAA
Leptin	ATCTTCCGGGCCCTTGTCTA	CAGAGTCAGATGGAGCTGGC

*Pax7* paired box 7*, MHC* myosin heavy chain, *GPADH* glyceraldehyde-3-phosphate dehydrogenase, *ACTB* actin beta. *TP53* tumor protein p53, *TP53I3* tumor protein p53 inducible protein 3, *TP53RK* TP53 regulating kinase, *MYC MYC* proto-oncogene, *PTEN* phosphatase and tensin homolog, *EGFR* epidermal growth factor receptor, *TERT* telomerase reverse transcriptase, *DKC1* dyskerin pseudouridine synthase 1*, CD73* 5’-nucleotidase ecto, *CD105* endoglin, *PPARγ* peroxisome proliferator activated receptor gamma, *C/EBPα* CCAAT enhancer binding protein alpha, *LPL* lipoprotein lipase.

### Optimization of the myogenic differentiation medium (DM)

**Fig. 3:**
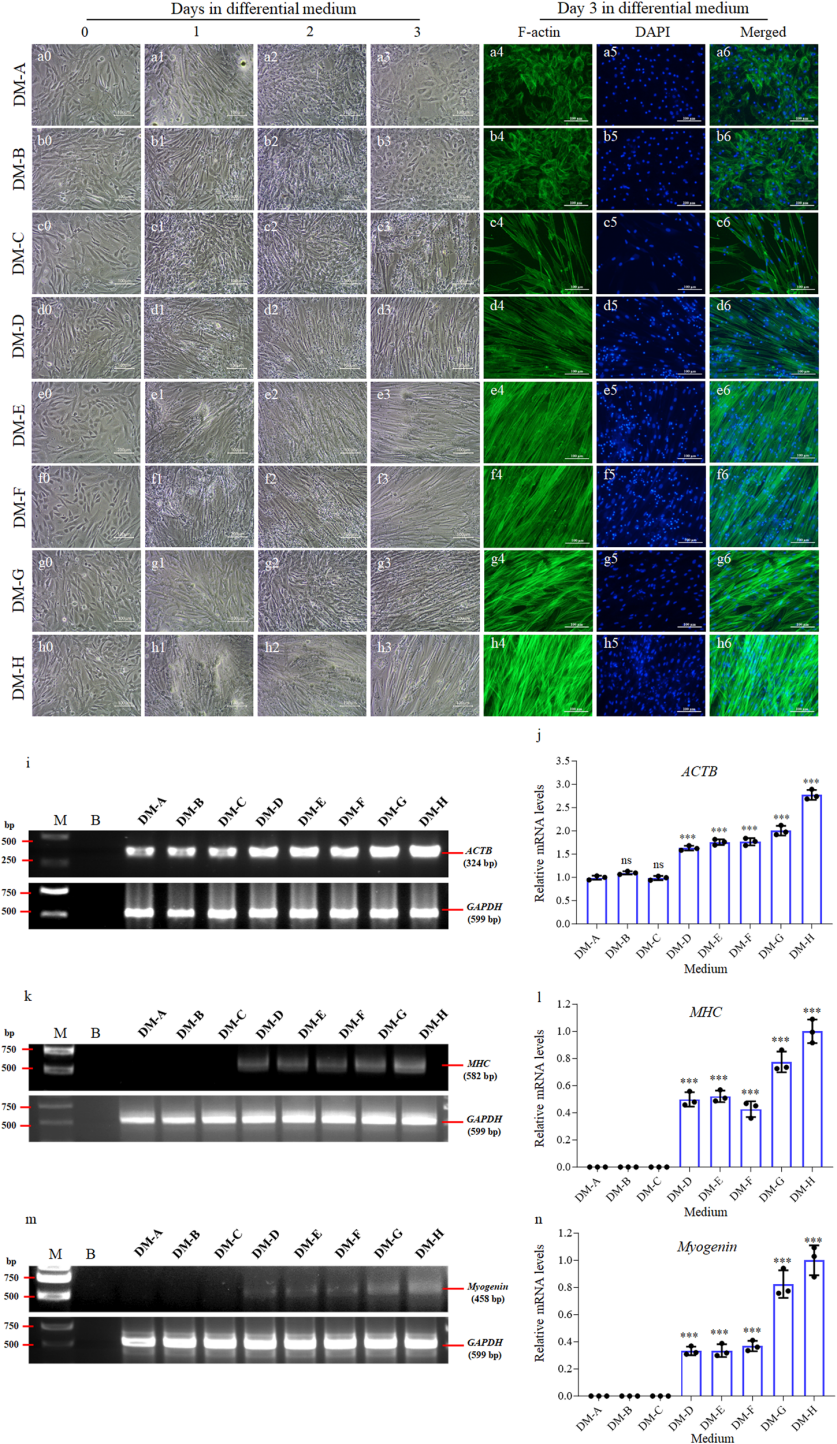
Optimization of the myogenic differentiation medium. **a**–**h** EfMS cells cultured in different differentiation media of DM-A ~ DM-H for 0–3 days (left four light micrographs) and phalloidin (green) and DAPI (blue) staining on day 3 (right three fluorescent micrographs), respectively. Scale bar: 100 μm. **i** Agarose gel electrophoresis results of semi-quantitative RT-PCR amplification of *ACTB* and *GAPDH* from the EfMS cells cultured in DM-A ~ DM-H, respectively. **j** Statistical results of gray values of each band in (**i**), error bars indicate SD, *n* = 3. **k** Agarose gel electrophoresis results of semi-quantitative RT-PCR amplification of *MHC* and *GAPDH* from the EfMS cells cultured in DM-A ~ DM-H. **l** Statistical results of gray values of each band in (**j**), error bars indicate SD, *n* = 3. **m** Agarose gel electrophoresis results of semi-quantitative RT-PCR amplification of *Myogenin* and *GAPDH* from the EfMS cells cultured in DM-A ~ DM-H. **n** Statistical results of gray values of each band in (**l**), error bars indicate SD, *n* = 3. M, DNA marker; B, blank control. Triple asterisks (***) stand for *p* < 0.001.

To induce the myogenic differentiation of EfMS cells, the low-concentration-horse-serum medium were initially attempted but produced low differentiation efficiency. Thus, we formulated eight kinds of different myogenic differentiation media, designated as DM-A ~ DM-H as detailed in Table 3, by adding varied concentrations of vitamin C, vitamin D, insulin, horse serum, and mesenchymal stem cell-specific FBS into the basic medium of L-15 or MEM, and compared their myogenic differentiation efficiencies in the EfMS cells. Among them, DM-H was found to be the most effective DM (Fig. 3). In DM-H, the majority of EfMS cells fused to each other and formed multinucleated myotubes and expressed higher levels of fibrous actin (Fig. 3a–h). This was further validated by semi-quantitative RT-PCR analysis of the mRNA expression of F-actin gene (i.e., *ACTB*) (Fig. 3i, j). Additionally, the expression levels of *MHC* (*Myosin Heavy Chain*) and *Myogenin*, markers of terminal muscle cell differentiation^30^, were also significantly up-regulated in the EfMS cells cultured in DM-H, indicating an enhanced myogenic differentiation efficiency (Fig. 3k–n).

**Table 3:** Formulas of the 8 kinds of myogenic differentiation media (DM)

Medium	Formula^a^
DM-A	97% MEM + 2% HS
DM-B	97% L-15 + 2% HS
DM-C	97% MEM + 2% FBS
DM-D	97% L-15 + 2% FBS
DM-E	97% L-15 + 2% FBS + 10 μg mL^-1^ insulin
DM-F	97% L-15 + 2% FBS + 200 μM vitamin C
DM-G	97% L-15 + 2% FBS + 100 nM vitamin D
DM-H	97% L-15 + 2% FBS + 10 μg mL^-1^ insulin + 200 μM vitamin C + 100 nM vitamin D

*HS* horse serum, *FBS* fetal bovine serum, quantified for mesenchymal stem cells (BI, Israel, Cat. 04-400).

^a^All the DMs were supplemented with 1% penicillin-streptomycin mixture.

### Proliferation and myogenic differentiation of EfMS cells on 3D edible microcarriers and production of cell-cultured fish meat

**Fig. 4:**
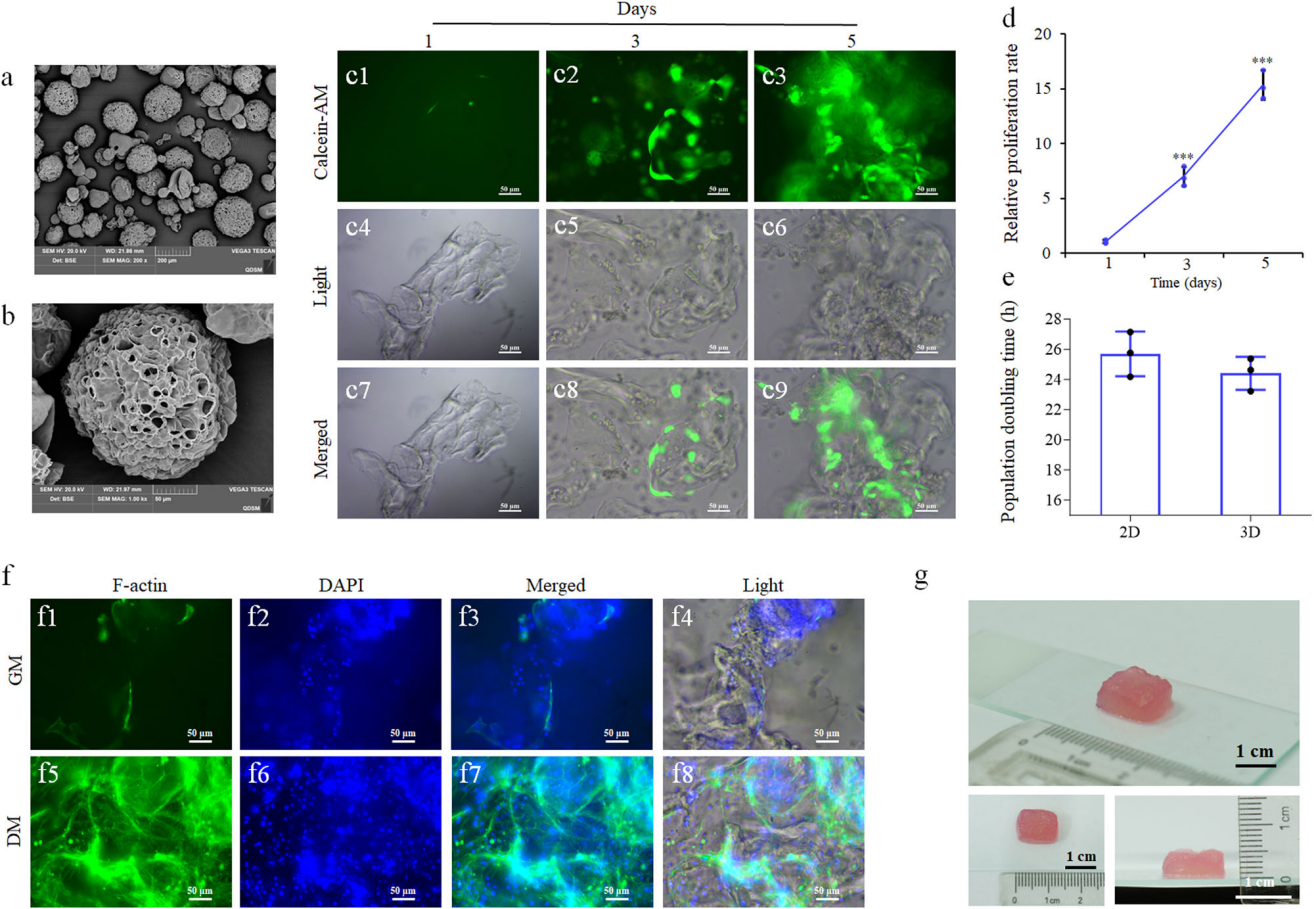
Proliferation and myogenic differentiation of EfMS cells on edible microcarriers and production of cell-cultured fish meat. **a** Micrograph of edible 3D microcarriers under 200× scanning electron microscopy. Scale bar: 200 μm. **b** Micrograph of edible 3D microcarriers under 1000× scanning electron microscopy. Scale bar: 50 μm. **c** Calcein-AM (green) staining results of EfMS cells growing on the microcarriers for 1, 3, and 5 days, respectively. Scale bar: 50 μm. **d** Growth curves of EfMS cells cultured in 3D microcarriers for 5 days, respectively. Cells were counted every two days. Error bars indicate SD, *n* = 3. Triple asterisks (***) stand for *p* < 0.001. **e** Calculated population doubling times of EfMS cells in 2D and 3D culture. **f** Phalloidin (green) and DAPI (blue) staining of the EfMS cells cultured in differentiation medium (DM-H) or growth medium (SCM-G) for 3 days, respectively. Scale bar: 50 μm. **g** Cell-cultured fish meat. Scale bar: 1 cm.

To acquire a sufficient number of EfMS cells for the production of cell-cultured fish meat, edible gelatin-sourced microcarriers was employed as a cell expansion scaffold (Fig. 4a, b). About 5 × 10^6^ EfMS cells were seeded onto 20 mg microcarriers to assess the proliferation capability of EfMS cells. Results showed that the cells could thrive on the microcarriers and exhibit a 15.3-fold increase in cell number over a 5-day culture period (Fig. 4c, d), with a higher growth rate (~24.39 h/PDT) compared to 2D culture (~26.15 h/PDT) (Fig. 4e).

When the EfMS cells reached a high cell density on microcarriers, the myogenic differentiation was induced by medium replacement from SCM-G to DM-H, and myogenic differentiation of EfMS cells was confirmed by Phalloidin staining. The results obtained revealed that in DM-H medium, the cells exhibited a slender, fibroblast-like morphology and expressed higher levels of F-actin. In contrast, cells in the growth medium were much shorter in shape with lower F-actin expression (Fig. 4f). This finding suggested that EfMS cells could differentiate on microcarriers. When 5 × 10^7^ cells were seeded onto 0.2 g microcarriers, 7.5 × 10^8^ cells could be obtained 5 days later. After switched to DM-H medium to induce myogenic differentiation and then harvested on the third day post differentiation induction, centimeter-scale cell-cultured fish meat could be produced with a wet weight of 0.65 g (Fig. 4g).

### Production and myogenic differentiation of scaffold-free EfMS cell balls and amino acid content analysis

**Fig. 5:**
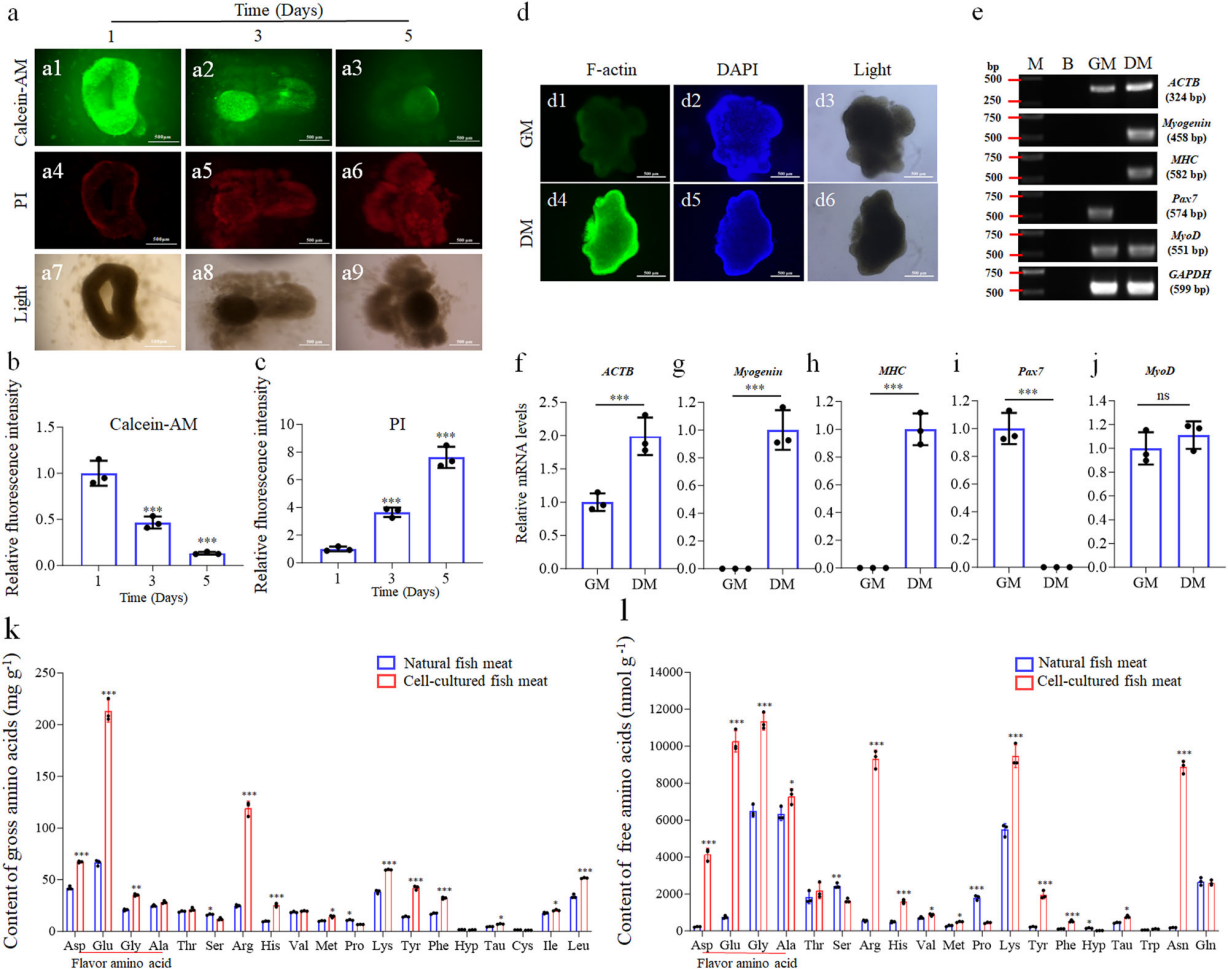
Production, myogenic differentiation and amino acid content analysis of scaffold-free EfMS cell balls. **a** Calcein-AM (green) and propidium iodide (PI, red) staining of EfMS cell balls in blunt-bottomed ultralow-binding culture plates. Scale bar: 500 μm. **b** Quantitative analysis of Calcein-AM (green) fluorescence intensity, error bars indicate SD, *n* = 3. **c** Quantitative analysis of PI (red) fluorescence intensity, error bars indicate SD, *n* = 3. **d** Myogenic differentiation of one-day EfMS cell balls, stained by phalloidin (green) and DAPI (blue) at the 3rd day post induction. Scale bar: 500 μm. **e** Agarose gel electrophoresis results of semi-quantitative RT-PCR amplification of *ACTB*, *Myogenin*, *MHC*, *Pax7*, *MyoD* and *GAPDH* from the EfMS cell balls cultured in DM-H and GM, respectively. M, DNA marker; B, blank control. **f**–**j** Statistical results of gray values of each band in e, error bars indicate SD, *n* = 3. Triple asterisks (***) stand for *p* < 0.001. ns stands for *p* > 0.05. **k** Analysis results of the composition and content of gross amino acids. **l** Analysis results of the composition and content of free amino acids. Error bars indicate SD, *n* = 3. Asterisk (*) stands for *p* < 0.05, Double asterisks (**) stand for *p* < 0.01, Triple asterisks (***) stand for *p* < 0.001.

Scaffold-free EfMS cell balls could be produced by seeding the cells into a 12-well blunt-bottomed ultralow-binding culture plate at a density of 2 × 10^8^ cells/well and then incubated for 24 h (Fig. 5a). The EfMS cells in each well spontaneously formed a cell ball. Results of the cell viability assay by Calcein-AM and PI staining indicated that, initially, the cell ball at day 1 exhibited high viability, with most cells being Calcein-AM positive and only a few being PI positive. However, their viability declined over time, and by day 5, only a few cells remained alive, indicating limited cell survival (Fig. 5a–c). The one-day EfMS cell balls were used to evaluate the myogenic differentiation potential, it was found that, after the growth medium (SCM-G, Table 1) was replaced with myogenic differentiation medium (DM-H, Table 3), efficient myogenic differentiation and myofiber formation within the EfMS cell ball could be detected on the third day post differentiation induction, evidenced by higher levels of F-actin expression in the cell balls cultured in DM-H compared to those in the SCM-G (Fig. 5d). Additionally, semi-quantitative RT-PCR results for *ACTB*, *Myogenin*, *MHC*, *Pax7*, and *MyoD* genes further validated the myogenic differentiation potential of the cell ball (Fig. 5e–j). These findings suggest that while scaffold-free 3D-cultured cell balls are capable of undergoing myogenic differentiation, their limited survival and proliferation potential will restrict their direct applicability for large-scale scaffold-free cell-cultured fish meat production.

To evaluate the nutritional value of the cell-cultured fish meat, the composition and content of both gross and free amino acids in the scaffold-free EfMS cell-cultured fish meat were analyzed and compared with those of natural fish meat, respectively. Our findings indicated that the EfMS cell-cultured fish meat contained higher levels of flavor amino acids including glutamic acid, asparagine, glycine and alanine than natural fish meat (Fig. 5k, l). Especially the delicate amino acid of glutamic acid, its gross and free contents were 3.21 and 13.73 folds of the natural fish meat, respectively. In addition, the gross and free amino acid contents of the basic arginine in cell-cultured fish meat were found to be 4.80 and 16.93 folds of the natural fish meat, respectively. This significant increase in arginine could potentially enhance the nutritional profile of the cell-cultured fish meat, benefit cardiovascular health and enhance immunity. This suggests that cell-cultured fish meat may have a more favorable taste and nutrition than natural fish meat and could be more appealing to consumers. However, rigorous sensory evaluation studies are needed in the future to scientifically assess consumer preferences and validate these potential improvements in taste.

### Trans-differentiation of EfMS cells into adipocytes and production of fat-containing cell-cultured fish meat

**Fig. 6:**
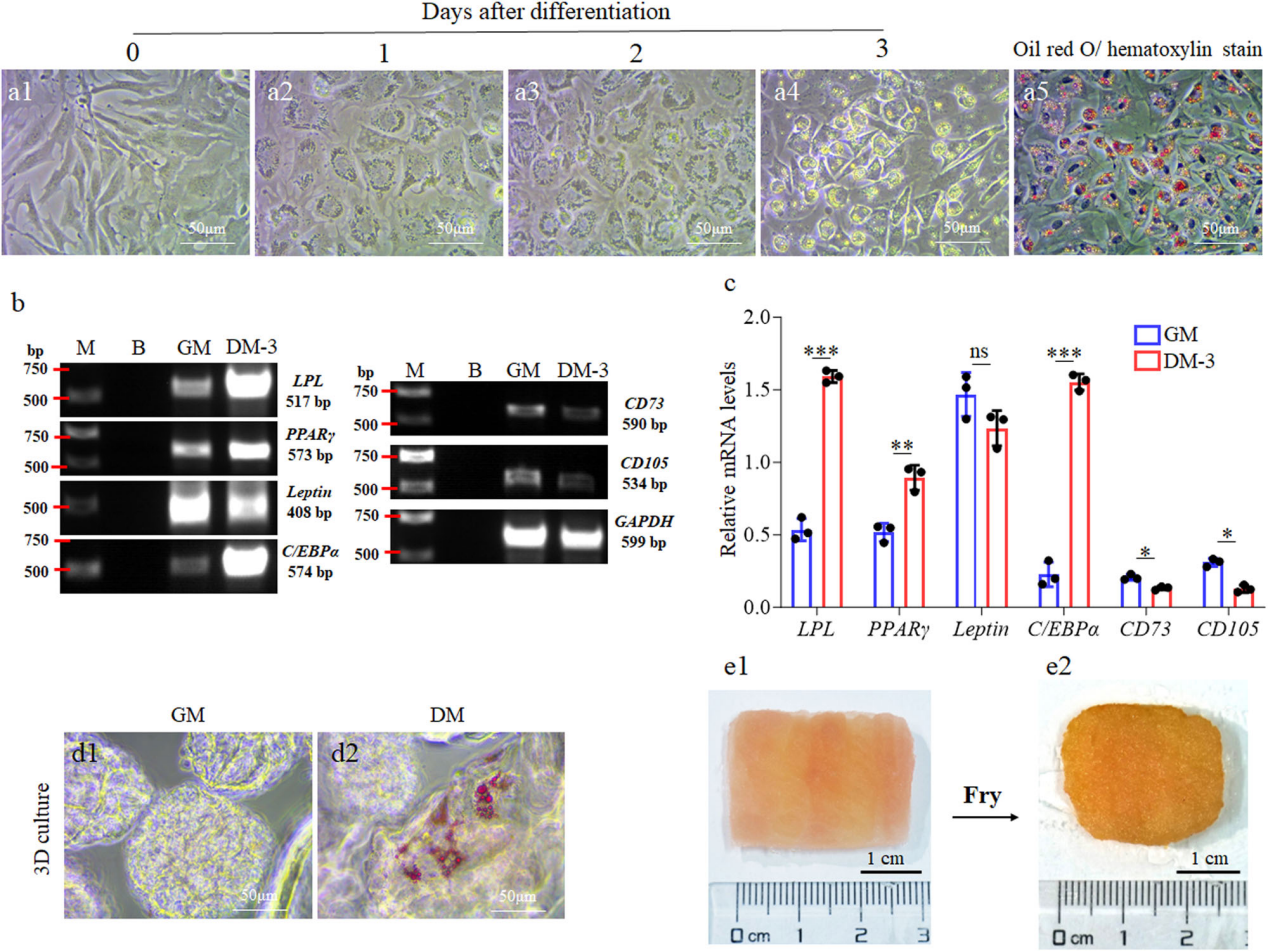
Trans-differentiation of EfMS cells to adipocytes. **a1** Light micrograph of EfMS cells cultured in growth medium. **a2**–**a4** Light micrographs of EfMS cells trans-differentiated for 1, 2 and 3 days, respectively. **a5** Light micrograph of EfMS cells stained with Oil Red O (red) and hematoxylin (blue) on day 3 of trans-differentiation. **b** Agarose gel electrophoresis results of semi-quantitative RT-PCR amplification of the adipogenic differentiation genes of *LPL*, *PPARγ*, *Leptin* and *C/EBPα*, the preadipocytes marker genes of *CD73* and *CD105*, and the internal reference gene of *GAPDH*, from the EfMS cells cultured in adipogenic trans-differentiation medium on day 3 (DM-3) or growth medium (GM). M, marker; B, blank. **c** Statistical results of gray values of each band in (**b**), error bars indicate SD, *n* = 3. Asterisk (*), *p* < 0.05. Double asterisks (**), *p* < 0.01. Triple asterisks (***), *p* < 0.001. ns *p* > 0.05. **d1–d2** Light micrographs of EfMS cells on microcarriers stained with Oil Red O (red) on day 3 in DM or GM medium. **e1** Fat-containing cell-cultured fish meat. Muscle fibers, red. Fat tissues, white. Scale bar: 1 cm. **e2** Fried fat-containing cell-cultured fish meat. Scale bar: 1 cm.

The aroma of meat is highly dependent on the content and distribution of fat tissue. Thus, we sought the possibility to trans-differentiate EfMS cells into adipocytes. Mammalian adipogenesis is reported to be induced by the use of insulin, dexamethasone, 3-isobutyl 1-methylxanthine (IBMX), palmitic acid, and oleic acid^31^. Based on this, we designed and tested an adipogenic trans-differentiation medium containing 1 μM Dexamethasone, 0.45 mM IBMX, 10 μg mL^−1^ Insulin, 500 μM palmitic acid, 500 μM oleic acid and 20% FBS. It was found that the EfMS cells could be successfully induced by the above-mentioned adipogenic trans-differentiation medium to differentiate into mature adipocytes, characterized by the formation of numerous lipid droplets within the cells, which could be stained red with Oil Red O (Fig. 6a). Gene expression analysis showed that the transcriptional expression of the adipogenic differentiation genes of *LPL*, *PPARγ* and *C/EBPα* were significantly upregulated (Fig. 6b, c), about 2.87, 1.75 and 6.34 folds of the uninduced EfMS cells, respectively. In contrast, after the adipogenic trans-differentiation induction, the transcriptional expression levels of the preadipocytes markers of *CD73* and *CD105*, initially at a very low level in growth medium, were further downregulated instead (Fig. 6b, c). These data suggested that the EfMS cells also retained the characteristics of preadipocytes and could be trans-differentiated into adipocytes. It was also found that the EfMS cells growing on the edible microcarriers could be induced trans-differentiated into adipocytes (Fig. 6d). Finally, the fat-containing cell-cultured fish meat was successfully produced by separately inducing the myogenic differentiation and adipogenic trans-differentiation of the EfMS cells on the edible microcarriers, and then harvesting the microcarriers carrying EfMS cells-derived myofibers or adipocytes on the third day of differentiation induction (Fig. 6e).

To evaluate the flavor profile of fat-containing cell-cultured fish meat, we performed a qualitative and quantitative analysis of volatile compounds in both cell-cultured and natural fish meat using Gas Chromatography-Mass Spectrometry (GC-MS). As shown in Supplementary Table 1, a total of 14 kinds of volatile compounds have been detected from the natural fish meat, while 22 kinds from the cell-cultured fish meat, and a total of 31 kinds from both samples. The detected 31 kinds of volatile compounds can be grouped into aldehydes (3), ketones (3), alcohols (8), acids (2), hydrocarbons (4), esters (3), and others (8). Notably, much more alcohols (7) were detected from the cell-cultured fish meat than those in the natural fish meat (2 alcohols), and 3 kinds of esters were detected from the cell-cultured fish meat whereas no esters from the natural fish meat, suggesting a richer aroma potential for the fat-containing cell-cultured fish meat in comparison with natural fish meat. Obviously, this can be attributed to the introduction of the trans-differentiated adipocytes.

## Discussion

Cell-cultured fish meat is an emerging innovative food source that may potentially alleviate fishing pressure and promote more sustainable farming practices^32,33^. In this study, we had established and characterized a spontaneously immortalized, marine fish muscle stem cell line (EfMS) from the brown-marbled grouper (*E. fuscoguttatus*) with undiminished stemness even after 50 passages. Of note, the non-proteinous essential amino acid of taurine was found to play an important role in maintaining the stemness of EfMS cells and could serve as a substitute for fish muscle extract in the stem cell growth medium. Moreover, the culture conditions for active proliferation and efficient myogenic differentiation and adipogenic trans-differentiation of EfMS cells in both 2D and 3D culture systems had been successfully developed and used to produce fat-containing cell-cultured fish meat.

The production of cultured meat begins with the isolation of muscle stem cells^4^. Up to now, the isolation of muscle stem cells from cattle, pigs, and chickens have been well developed, allowing for large quantities of muscle stem cells to be obtained through FACS-sorting^34,35^, but the proliferation and differentiation capacities of the isolated muscle stem cells is limited, and always lost their stemness quickly thereafter^13,36^. Although the Notch and p38 signaling pathways are found to be critical for maintaining the stemness, there are no immortalized mammalian and bird muscle stem cell lines available yet^36,37^, necessitating repeated isolation and purification of the muscle stem cells from adult tissues, increasing the cost of large-scale cell-cultured meat production. In contrast, studies on cell-cultured fish meat are far behind those in cattle, pigs, and chickens due to the lacking of appropriate muscle stem cell lines. The fish exhibit minimal senescence and continuous growth throughout their lives, largely due to high telomerase activity in nearly all fish tissues^38^. Similar results were obtained in the adult tissues and isolated EfMS cells of brown-marbled grouper in this study. One possible explanation for the relatively higher telomerase activity in the EfMS cells than the tested adult tissues is that, the EfMS cell line is an enriched cell population of muscle stem cells. This characteristic makes fish cell lines more likely to undergo spontaneous immortalization^39^, suggesting that establishing immortalized muscle stem cell lines from fish is more advantageous than from mammals, thereby offering better prospects for industrial-scale production of cell-cultured fish meat. Xu et al. isolated muscle stem cells from large yellow croaker (*Larimichthys crocea*), but their stemness was maintained only to passage 3 with approximately 43% of the cells were positive for Pax7^25^. However, they failed to develop a stable, immortalized fish muscle stem cell line. Nutrient insufficient in the culture medium may cause differentiation during extended in vitro culture^40^, indicating that enhancing nutrient content could help maintain the stemness. In our study, we have maintained the stemness of EfMS cells in vitro for at least 50 passages using SCM-A medium, which contains 69% L-15 medium, 20% FBS, 20 ng mL^−1^ bFGF, 20 ng mL^−1^ EGF, 10% muscle extract, and 1% antibiotics. These results indicated that we had established a spontaneously immortalized muscle stem cell line from brown-marbled grouper, representing a critical step forward in the production of cell-cultured fish meat.

The genetic stability and safety of seed cells used in the production of cell-cultured meat are critical for ensuring reliability and consumer acceptance. Induced pluripotent stem cells or CRISPR-immortalized muscle cells can proliferate indefinitely^1,41^. However, genetic modifications may cause off-target effects, raising concerns about consumer acceptance^42^. An alternative approach is spontaneous immortalization. During cell immortalization, the inactivation or dysfunction of the tumor suppressor protein p53 often signifies a cancerous cell. This process is usually accompanied by the up-regulation of EGFR, PTEN, and MYC genes^42^. In our study, we found that after the spontaneous immortalization of EfMS cells, only the MYC gene was down-regulated, while DKC1, a gene related to the stability and activity of telomerase, was up-regulated. These findings suggest that EfMS cell lines exhibit genetic stability. This genetic stability underscored the potential and safety of EfMS cells as an ideal seed cell for cell-cultured fish meat production.

Taurine, a nonproteinogenic β-aminosulfonic acid, is predominantly found in fish and seafood. For example, sardine (*Sardina pilchardus*) contains approximately 147 mg of taurine per 100 g meat, while tuna (*Thunnus spp*) has a higher taurine content, up to 332 mg per 100 g^43^. In comparison, human muscle tissue contains taurine in the range of about 27.5 to 67.6 mg per 100 g^44^. It is found that taurine plays a significant role in cellular redox homeostasis and skeletal muscle function. It can counteract lipid peroxidation and enhance cellular antioxidant defense, particularly in response to inflammation, highlighting its significance in cellular health^28^. Additionally, taurine can improve the proliferation and differentiation of neural stem cells, underscoring its potential in various stem cell cultures, including the muscle stem cells of course^45^. Similarly, in our study, taurine was found to be crucial in maintaining the stemness of EfMS cells, evidenced by its capability to serve as a substitute to fish muscle extract in the growth medium of SCM-A, thus, certainly a great benefit for the sustainability and low cost of cell-cultured fish meat production. As for the capability of taurine to replace the fish muscle extract, one possible explanation is that, except for taurine, all the other nutrients in the fish muscle extract, like free amino acids, saccharides and lipids, can be replenished from the L-15 medium.

A big challenge in cell-cultured fish meat production is the myogenic differentiation of muscle stem cells. It was found in this study that the traditional myogenic differentiation medium containing low concentration of horse serum, which was efficient for mammalian muscle stem cells^46^, were not applicable to fish. This necessitated exploring alternative differentiation strategies. Accumulating evidences have showed that vitamin C and D played a pivotal role in this context. Vitamin C could promote skeletal muscle growth and slow down skeletal muscle atrophy through antioxidant and anti-inflammatory effects^47,48^. The vitamin C transporter SVCT2, preferentially expressed in slow muscle fibers, was influential during early myogenesis, indicating a broader role of vitamin C in skeletal muscle development^49^. Vitamin D, essential for skeletal muscle, could modulate cell cycle, proliferation, differentiation, and apoptosis. Deficiency in vitamin D could induce muscle fiber atrophy^50,51^. Skeletal muscle is the main organ for insulin-induced glucose metabolism, and insulin is vital for muscle synthesis^52^. Previous studies have showed that vitamin C, vitamin D and insulin could promote the myogenic differentiation of mouse myoblast cell line (C2C12)^53–56^. Given these roles, we hypothesized that vitamin C and D, along with insulin, might similarly promote the myogenic differentiation of fish muscle stem cells. Our results aligned with these findings, showing that EfMS cells differentiated most effectively in the medium supplemented with vitamin C, vitamin D and insulin. This was evidenced by the formation of multinucleated myotubes, elevated F-actin levels, and upregulated mRNA expression of key muscle markers such as MHC and Myogenin. In another word, the use of vitamins in the myogenic differentiation medium has extended beyond their nutritional functions, but modulated the cell growth and differentiation, highlighting their potential in enhancing cell-cultured fish meat quality^57^.

Nutrition is crucial for both natural and cell-cultured meat^58^. The amino acid analysis results revealed that scaffold-free cell-cultured fish meat had a higher abundance of flavor amino acids (glutamic acid, asparagine, glycine and alanine) and basic amino acid (arginine) than the natural fish meat. This may be attributed to the higher concentrations of amino acids in the culture medium and the controlled in vitro culture environment, which facilitated an efficient nutrient uptake. Anyway, this has heralded a good way for us to enhance the palatability and acceptance of cell-cultured fish meat among consumers in future.

Adipose tissues are essential for regulating the flavor, taste and nutrition of fish meat. Currently, no immortalized fish preadipocytes line has been established, and their culture stops at the primary stage^59,60^. Interestingly, we found that the EfMS cells also transcriptionally expressed two marker genes of *CD73* and *CD105* at a relatively low level. This is a remarkable thing given that these two genes are predominantly expressed in preadipocytes and are critical for adipogenesis and mesenchymal stem cell properties^61,62^. This finding suggests that fish muscle stem cells may have higher plasticity than those of mammals. Subsequent adipogenic trans-differentiation experiments further confirmed the potential of EfMS cells to differentiate into mature adipocytes. Therefore, the EfMS cell line could serve as a viable seed cell substitute for preadipocytes in the production of fat-containing cell-cultured fish meat, to enhance the sensory qualities of cell-cultured fish meat.

While our research marks significant progress, further studies are needed to optimize the scale up production of cell-cultured fish meat for commercialization. For example, to assess and improve the sensory properties including texture, flavor, and aroma which will determine the consumer acceptability, to reduce the production cost through cheaper culture medium and improved proliferation and differentiation efficiency, are all needed and essential in the future work. Moreover, avoiding ethical concerns about the use of animal-derived materials in culture media by developing serum-free or plant-based alternatives are also necessary^63^.

In conclusion, we have successfully established a stable, spontaneously immortalized muscle stem cell line from brown-marbled grouper (EfMS) with undiminished stemness. This EfMS cell line will provide us an ideal seed cell line for cell-cultured fish meat production. While significant challenges remain, we are optimistic that cell-cultured fish meat can become a viable and sustainable alternative to conventional fish meat in the near future.

## Materials and methods

### Primary cell culture and subculture

Healthy brown-marbled groupers (*E. fuscoguttatus*) with 3 ± 0.5 cm in length were purchased from a fish farm in Zhanjiang (Guangdong, China). The fish were pretreated in boiling-sterilized seawater containing 1000 IU mL^−1^ penicillin and 1000 μg mL^−1^ streptomycin for 24 h. The fish were then anesthetized by MS-222 (Sigma, USA) and disinfected by immersing in 75% ethanol for 2 to 3 min. The muscle tissues above the lateral line of the fish were aseptically dissected with a surgical scalpel and washed 3 times in D-hank’s solution (8 g L^-1^ NaCl, 0.4 g L^-1^ KCl, 0.06 g L^-1^ KH_2_PO_4_, 0.08 g L^-1^ Na_2_HPO_4_·12H_2_O and 0.35 g L^-1^ NaHCO_3_, pH 7.0) supplemented with 10% penicillin-streptomycin mixture in a 50-mL centrifuge tube (Corning, USA) on ice. After that, the muscle tissues were transferred to a petri dish and cut into pieces with ophthalmic scissors, and then transferred to a 50-mL centrifuge tube again. Next, the tissue pieces were subjected to 2 mL L-15 medium supplemented with 2.5 mM CaCl_2_, 1.25 mg mL^-1^ dispase type II (Roche, Switzerland) and 5 mg mL^-1^ collagenase D (Roche, Switzerland) and digested for 1.5 h at 28 °C on a water bath shaker at 170 rpm. The digested suspension was first filtered through a 70 μm filter membrane (BD-Pharmingen, USA) to remove large tissue blocks and then rinsed with 12 mL L-15 medium (Gibco, USA). After filtration, the filtrate was collected and centrifuged at 300 × *g* for 5 min. The supernatant was discarded and the cell precipitate was resuspended in 2 mL fish muscle stem cell medium (SCM-A) as listed in Table 1. The cells were counted using a hemocytometer, and a total of 1 × 10^5^ cells per dish were seeded into a 3.5-cm cell culture dish (Corning, Cat. 353001, USA). The cells were incubated at 28 °C in a biochemical incubator without CO_2_. The medium was half changed every 48 h. The muscle stem cells were daily monitored and photographed under an inverted contrast phase microscope (Nikon, Japan).

Subsequently, the primary cell monolayer was sub-cultured using 0.0625% trypsin-EDTA solution and then a new marine fish muscle stem cell line of EfMS (*E. fuscoguttatus* muscle stem cell) was obtained. Cryopreservation of the cell line was conducted using a serum-free cryopreservation solution (Procell, China) at passages 5, 8, 10, 12, 15, and every 5 passages thereafter until passage 80. Two cryopreservation tubes (Coring, USA) were prepared during each cryopreservation: one for long-term storage and another thawed after one week to confirm viability. After passage 20, the concentration of trypsin-EDTA used for subculture was returned to 0.25%.

For the preparation of muscle extract, the adult brown-marbled grouper was anesthetized using MS-222. Once fully anesthetized, the fish was euthanized by a swift and forceful blow to the head using a hammer. Following euthanasia, the fish was descaled, and the skin was carefully removed. The dorsal muscle tissue was then dissected, ensuring that any bones, connective tissues, and skin remnants were removed. The extracted muscle tissues were rinsed with sterile PBS to remove blood and surface contaminants, then cut into small pieces for further processing. The muscle tissues of brown-marbled grouper were then homogenized in L-15 medium in a volume of 3 mL per g of tissue. After that, the homogenate was incubated in a 60 °C water bath for 1 h with inverted mixing every 10 min and then clarified by centrifugation at 8000  × *g* for 2 h at 4 °C. The supernatant was collected and adjusted to pH 7.0 with 0.1 g mL^−1^ NaHCO_3_, and sterilized by a 0.22 μm filter membrane (Sartorius, Germany). The protein content of the muscle extract was determined using the BCA Protein Assay Kit (Solarbio, China), with a concentration measured at 22.1 mg mL^−1^.

All animal studies were performed in accordance with the Ethical Guidelines for the Use and Care of Laboratory Animals and were approved by the Animal Care and Use Committee of Ocean University of China. We have complied with all relevant ethical regulations for animal use. The fish used for cell isolation were one-month-old females, while those used for muscle extract preparation were six-month-old females.

### DNA extraction and CO1 gene analysis

The species origin of EfMS cell line was determined by sequencing the mitochondrial *CO1* (cytochrome oxidase subunit 1) gene. Total genomic DNA of the EfMS cells at passage 20 was extracted using a DNA extraction kit (Tiangen, China) and used as a template to amply the *CO1* gene. The forward primer used was 5’-ATTGGCACCCTTTATCTTGTA-3’ and reverse primer was 5’-TGAGAGATAGCAGGGGGTTTTA-3’. The PCR amplification was carried out in a 25 μL volume containing 12.5 μL of 2×Taq Mix, 2.0 μL of template DNA (60 ng), 1.0 μL forward primer (10 μM) and 1.0 μL reverse primer (10 μM). The PCR reaction involved in an initial denaturation at 94 °C for 10 min, followed by 30 cycles of denaturation at 94 °C for 30 s, renaturation at 55 °C for 30 s, elongation at 72 °C for 30 s, and a final extension at 72 °C for 10 min. PCR products were analyzed by 1.0% agarose gel electrophoresis and then sequenced. The obtained sequences were aligned against known sequence of *E. fuscoguttatus CO1* gene (GenBank No. NC_020046.1) deposited in the NCBI database.

### Optimization of fish muscle stem cell medium (SCM)

To obtain an optimal SCM with a capability to support the active proliferation of EfMS cells along with undiminished stemness, this SCM was developed and optimized by successively analyzing the culture effects of varied concentrations of FBS (10% in SCM-E or 20% in SCM-A, B, C and D), grouper muscle extract (none in SCM-C, D and E or 10% in SCM-A and B) and growth factors (none in SCM-B, D and E or 20 ng mL^−1^ bFGF and 20 ng mL^−1^ EGF in SCM-A and C) on the EfMS cells. To do this, EfMS cell monolayers were prepared by seeding them into a 96-well culture plate (Corning, USA) at a density of 3 × 10^3^ cells/well. After incubation at 28 °C for 24 h, the old medium in each well was discarded and replaced with the tested 5 kinds of SCMs (A ~ E) as listed in Table 1, and continued to culture for another 4 days until confluency. After the addition of SCM, every 24 h, the cells in three wells were separately collected by trypsinization and counted with a hemocytometer and used to plot the growth curves for each tested SCM. The growth curve was plotted as EfMS cell number against culture time. The population doubling time (PDT) was calculated as follows: PDT = (ΔT×ln2) / [ln (Final cell number)–ln (Initial cell number)]

Screening of a substitute for fish muscle extract in SCM

The results previously obtained in this study have showed that the fish muscle extract supplemented in SCM played an important role in maintaining the stemness of EfMS cells. However, the use of muscle extract was labor- and time-consuming, with high-cost and undefined nutrient composition, thus unsuitable for the scale-up production of cell-cultured fish meat. To screen a substitute for fish muscle extract, we first analyzed the composition and content of the free amino acids in the muscle extract derived from the adult grouper. In brief, 2 mL of muscle extract was mixed with 2 mL of 5% sulfosalicylic acid solution and then centrifuged at 8000 *g* for 10 min at 4 °C. The supernatant was then filtered through a 0.22 μm water-based filter membrane into a vial. This sample was subsequently injected into a L-8900 amino acid analyzer (Hitachi, Japan) to determine the amino acid content. The analysis results revealed that the top two most abundant components in the muscle extract were taurine (554 μg mL^-1^) and PEA (240 μg mL^-1^) (Supplementary Fig. 2). Both of them are absent in L-15 medium.

Notably, taurine plays a significant role in skeletal muscle function, and PEA has been shown to modulate cellular autophagy, which can extend cell longevity^28,29^. Based on this, as listed in Table 1, SCM-F and SCM-G were designed by replacing the muscle extract in SCM-A with taurine and PEA at their measured contents in the fish muscle extract, respectively. The culture effects of SCM-F and SCM-G were analyzed as described previously.

### Chromosomal analysis

EfMS cells at passage 30 were used for chromosome analysis. In brief, the EfMS cells were seeded into 25-cm^2^ culture flasks and incubated at 28 °C for 24 h, and then the old medium was replaced with fresh medium containing 10 μg mL^-1^ colchicine (Solarbio, China). After 2 h of incubation, the cells were collected by trypsinization and centrifugation at 70 × *g* for 3 min, and then exposed to 5 mL 0.075 M KCL solution for 30 min. After hypotonic treatment, the tubes were centrifuged again at 70 × g for 3 min and the cell pellet was subsequently fixed in freshly prepared, ice-cold methanol–acetic acid (3:1) for 15 min. The cell pellet was then resuspended in the fixative and spread onto a glass slide. After air-drying the slides, they were stained with a 5% Giemsa staining solution (Sigma, USA) at pH 6.8 for 15–20 min. Once stained, the slides were rinsed, air-dried, and the chromosome numbers of 100 metaphase-stage cells were counted under a microscope (Leica, Germany).

### Semi-quantitative RT-PCR

Using semi-quantitative RT-PCR technology, the transcriptional expression levels of the muscle stemness marker gene (*Pax7*) in the EfMS cells cultured in various growth media during the process of passages 5 to 50 were monitored, the differential expressions of 8 kinds of oncogenesis-related genes (*TP53, TP53I3, TP53RK, MYC, PTEN, EGFR, TERT* and *DKC1*) in the EfMS cells between passages 20 and 80 were compared (Supplementary Fig. 3), and also, 3 kinds of myogenic differentiation-related genes (*MHC, Myogenin* and *ACTB*) (Fig. 3), 4 kinds of adipogenic differentiation-related genes (*PPARγ, C/EBPα, LPL* and *Leptin*) and 2 kinds of preadipocyte marker genes (*CD73* and *CD105*) (Fig. 6) in the EfMS cells before and after they were induced to myogenic differentiation or adipogenic trans-differentiation were analyzed, respectively. The total RNAs of the tested EfMS cells were extracted using a total RNA extraction kit (Tiangen, China) according to the manufacturer’s instruction. Then the total RNAs (1 μg) were reversely transcribed into cDNAs using ReverAidTM First Strand cDNA Synthesis kit (Thermo Fisher Scientific, USA) and used as PCR templates. All the tested genes and their gene-specific primers were listed in Table 2. All the target gene fragments were amplified using the same reaction condition as described previously for the *CO1* gene analysis. Semi-quantitative RT-PCR products were analyzed by 1.5% agarose gel electrophoresis, and gray scale analysis was performed by Image J software to quantitatively detect the differential gene expression.

Furthermore, we compared the transcriptional expression levels of *TERT* and *DKC1* in 11 kinds of tissues (skin, gallbladder, fat tissues, kidney, spleen, heart, gills, intestines, muscle, fin, and liver) and EfMS cells at passage 20 using the same method described above (Supplementary Fig. 4).

### Immunofluorescence staining

The expression of Pax7 and MyoD proteins in the EfMS cells were analyzed by immunofluorescence staining. In brief, the tested EfMS cells were fixed in immunostaining fixative (Beyotime, China) for 10 min and then permeabilized in 0.2% Triton X-100 (Sigma-Aldrich, USA) for 10 min. Next, the cells were blocked in blocking solution (Beyotime, China) for 1 h at room temperature. After that, the EfMS cells were incubated with the mixed primary antibodies of anti-Pax7 antibody (1:10, DSHB, clonePAX7-s, USA) and anti-MyoD antibody (1:100, Santa Cruz, sc-377460, USA) overnight at 4 °C. After triple washing in PBS (8.0 g L^−1^ NaCl, 0.2 g L^−1^ KCl, 1.44 g L^−1^ Na_2_HPO_4_⋅12H_2_O and 0.24 g L^−1^ KH_2_PO_4_, pH 7.0), the corresponding two secondary antibodies were added and incubated for another 1 h at room temperature. Both the two secondary antibodies were purchased from Thermo Fisher Scientific with Alexa Fluor 488 goat anti-mouse IgG1 (1:500) for Pax7 and Alexa Fluor 555 goat anti-mouse IgG2b (1:500) for MyoD. Finally, the cell nuclei were counterstained with DAPI (1:1000 in water, Sigma, USA). Images were taken with an inverted fluorescence microscope (Nikon, Japan).

### Optimization of myogenic differentiation medium

Commonly used myogenic differentiation medium for mammalian muscle stem cells are not suitable for fish muscle stem cells. Thus, a new myogenic differentiation medium specific for EfMS cells was developed in this study by optimizing the basic medium of L-15 or MEM supplemented with varied concentrations of vitamin C (Solarbio, China), vitamin D (Solarbio, China), insulin (Sigma-Aldrich, USA), horse serum (Absin, China) or FBS (special for mesenchymal stem cells, BD-Pharmingen, USA). A total of 8 kinds of different myogenic differentiation media, designated as DM-A ~ DM-H as listed in Table 3, had been prepared and compared in their myogenic differentiation efficiencies. To do this, the EfMS cells were seeded into 6-well cell culture plates (Corning, USA) at a density of 1.5 × 10^5^ cells/well in 2 mL medium of SCM-A and cultured until the cell confluency reached 90–95%. Then, the old medium was discarded and the cell monolayer was washed once with PBS. Next, the medium in each well was replaced with 2 mL differentiation media tested to induce the myogenic differentiation of EfMS cells. The differentiation status of the cells was morphologically recorded daily and analyzed by Phalloidin staining (F-actin in green).

### Phalloidin staining

Phalloidin staining was used to detect the F-actin formation in the EfMS cells after induced by different myogenic differentiation media. After 3 days of differentiation induction, the old medium was discarded and the cell monolayers were washed with PBS. Then, the cells were fixed with 4.0% paraformaldehyde for 10–30 min at room temperature, followed by another wash with PBS. Next, the cells were permeabilized by 0.1% Triton X-100 for 3–5 min at room temperature, and then washed with PBS. After that, phalloidin staining solution (Absin, China) was added into the wells and the cells were stained for 20 to 90 min, and then washed once with PBS. Then, the nuclei of EfMS cells were counterstained with DAPI, and then the cell monolayers were washed once again with PBS. Images were captured under an inverted fluorescence microscope (Nikon, Japan).

### Induction of adipogenic trans-differentiation of EfMS cells

To trans-differentiate the EfMS cells into adipocytes, an adipogenic trans-differentiation medium was designed based on the mammalian adipogenic differentiation medium^31^. This medium consisted of 1 μM dexamethasone, 0.45 mM 3-isobutyl-1-methylxanthine (IBMX), 10 μg mL^−1^ insulin, 500 μM palmitic acid, 500 μM oleic acid, and 20% FBS (BI, Israel, Cat. 04-400). To induce the adipogenic trans-differentiation, the EfMS cells were plated into 6-well culture plates at a density of 1 × 10^5^ cells per well and cultured in the adipogenic trans-differentiation medium for 3 days. The medium in each well was replaced every 2 days. Finally, the adipogenic differentiation effects of the induced EfMS cells were analyzed by Oil Red O staining.

### Oil Red O staining

The Oil red O working solution was prepared by diluting the stock solution (Solarbio, China) with ddH_2_O at a ratio of 3: 2 and then filtered with 0.45 μm membrane. The EfMS cells were firstly washed three times with PBS and then fixed with 4% paraformaldehyde at 28 °C for 20 min. Then the cells were washed with PBS again and rinsed with 60% isopropanol for 1 min. Next, the cells were covered with the filtered Oil Red O working solution in dark at 28 °C for 30 min. After that, the Oil Red O was removed and the cells were washed with PBS and then treated with hematoxylin (Beyotime, China) in dark at 28 °C for 5 min. At last, the cells were washed with PBS and photographed under an inverted phase contrast microscope.

### Calcein-AM and propidium iodide (PI) staining

Calcein acetoxymethyl ester (Calcein-AM) is a non-fluorescent compound that can penetrate live cells due to its lipophilicity. Inside live cells, Calcein-AM can be converted by esterase into fluorescent Calcein, which is retained within the cells due to its non-lipophilic nature, thereby marking them as viable with a green fluorescence. Propidium iodide (PI) is a red-fluorescent dye that cannot penetrate live cells because of its impermeability, but can enter dead or dying cells with damaged membranes, and bind to nucleic acids, and emit red fluorescence, marking these cells as non-viable. To monitor the viability of the EfMS cells during 3D culture, the old medium in the culture plate was removed and the Calcein-AM and PI (Beyotime, China) working solution (1 μM) diluted in L-15 medium was added into each well and incubated at 28 °C in the dark for 45 min. Next, the cells were washed once with PBS and fresh medium was added, and observed under an inverted fluorescence microscope.

### 3D cell culture on microcarriers and production of fat-free and fat-containing cell-cultured fish meats

Edible 3D microcarriers fabricated from squid gelatin were kindly provided by Qingdao Institute of Marine Bioresources for Nutrition & Health Innovation (China). For 3D culture, the microcarriers in powder form were first added into a 48-well ultralow-binding cell culture plate (Corning, USA) at an amount of 20 mg/well. Next, the EfMS cells were harvested by trypsinization and centrifugation, and then suspended in the taurine-containing growth medium of SCM-G (Table 1) to a concentration of 1 × 10^7^ cells mL^−1^, and seeded into the plates preloaded with microcarriers at a volume of 500 μL per well and incubated at 28 °C for the production of fat-free or fat-containing cell-cultured fish meat. The medium in each well was changed daily.

To assess the proliferation of EfMS cells, the 3D microcarriers with attached cells were first harvested at days 1, 3, and 5 by centrifugation, and then washed with 2 mL of PBS. After that, 1 mL of 0.25% trypsin-EDTA was added, and the mixture was incubated at 28 °C for 1 min to detach the cells from the microcarriers. The detached cells (with a diameter of 10–20 μm) were efficiently separated from the microcarriers (with a size of 150–300 μm in diameter) by filtering the cell-microcarrier suspension through a 70 μm filter membrane. Following filtration, the cells were counted using a hemocytometer. The relative proliferation rate was calculated as the ratio of the number of cells counted to the number of cells on day 1.

To produce the fat-free cell-cultured fish meat, the EfMS cells were allowed to proliferate on microcarriers for 5 days, then the SCM-G medium was replaced with the myogenic differentiation medium of DM-H (Table 3) and the myogenic differentiation of the EfMS cells on microcarriers was induced. On the third day of differentiation induction, after the removal of medium, a total of 10 wells of microcarriers carrying EfMS myofibers were harvested and used to produce the centimeter-scale cell-cultured fish meat (Fig. 4).

To produce the fat-containing cell-cultured fish meat, the EfMS cells were first allowed to proliferate on microcarriers for 5 days, and then, the SCM-G media in 12 wells were replaced with the myogenic differentiation medium of DM-H (Table 3), respectively, whereas the SCM-G media in another 8 wells were replaced with the adipogenic trans-differentiation medium, and both myogenic and adipogenic differentiation of the EfMS cells were separately induced as described previously. On the third day of differentiation induction, all the 12 wells of microcarriers carrying EfMS myofibers and all the 8 wells of microcarriers carrying EfMS cell-derived adipocytes were harvested, respectively, and used to produce the fat-containing cell-cultured fish meat. In detail, the 12 wells of microcarriers carrying EfMS-derived myofibers were evenly divided into 3 portions, and the 8 wells of microcarriers carrying EfMS-derived adipocytes were divided into 2 portions. Next, these 5 portions were then arranged alternately in a muscle-fat-muscle pattern to produce the fat-containing cell-cultured fish meat (Fig. 6).

### Production of scaffold-free cell-cultured fish meat and its amino acid content analysis

For the production of scaffold-free cell-cultured fish meat, the EfMS cells in SCM-G medium (Table 1) were seeded into a 12-well blunt-bottomed ultralow-binding culture plate at a density of 2 × 10^8^ cells/well (Corning, USA). After 24 h of culture, the cells in each well spontaneously formed a cell ball. Next, the SCM-G medium in each well was replaced with myogenic differentiation medium of DM-H (Table 3) to induce the myogenic differentiation and myofiber formation within the EfMS cell ball. On the third day of differentiation induction, the differentiated cell balls were collected to produce the scaffold-free cell-cultured fish meat (Fig. 5).

To evaluate the nutritional value of cell-cultured fish meat, both the gross and free amino acid contents of the scaffold-free cell-cultured fish meats were analyzed and compared with those of natural fish meat, respectively (Fig. 5). For the gross amino acid content analysis, 100 mg of the scaffold-free cell-cultured fish meats or natural fish meat (i.e., grouper muscle tissue), were collected or dissected and then transferred to a 25-mL ampoule. After 10 mL of 6 M HCl (containing 5‰ mercaptoethanol) was added, the ampoule was subjected to hydrolysis at 110 °C for 22 h and then sealed under blowing nitrogen gas. The hydrolysate was then diluted in 0.02 M HCl to a final volume of 50 mL and then 1 mL of the hydrolysate was taken out and dried by blowing nitrogen gas. Next, 3–5 drops of ultrapure water were added and dried again to remove residual acid. Repeated this process three times, and then evaporated the solution to dryness. Finally, reconstituted the dried residue with 2 mL of 0.02 M HCl. Following reconstitution, filtered the solution through a 0.22 μm water-based filter membrane into a vial and injected it into a L-8900 amino acid analyzer (Hitachi, Tokyo, Japan) for the gross amino acid content analysis.

For the free amino acid content analysis, 100 mg of the scaffold-free cell-cultured fish meats or natural fish meat (i.e., grouper muscle tissue), were collected or dissected, respectively, and then transferred to a 2-mL Eppendorf tube. After adding two small steel balls and 1000 μL extraction solution (acetonitrile: methanol: water = 2: 2: 1, precooled at −40 °C and contained isotopically-labeled internal standard mixture), the sample was vortexed for 30 s, homogenized at 40 Hz for 4 min, and sonicated for 5 min in ice-water bath. The homogenate and sonicate circle were repeated for 3 times, followed by incubation at −40 °C for 1 h and centrifugation at 13,800 *g* for 15 min at 4 °C. A 100 μL aliquot of the clear supernatant was transferred to an auto-sampler vial for UHPLC-MS/MS analysis, performed by Shanghai Biotree Biotech co., LTD.

### Flavor measurement

Flavor analysis of cell-cultured fish meat and natural fish meat were analyzed using Gas Chromatography-Mass Spectrometry (GC-MS) (Thermo Fisher Scientific, USA). Volatile compounds from the fish samples were extracted using chromatography-grade acetone, followed by qualitative and quantitative analysis based on the ion fragmentation spectra obtained. For sample preparation, 2 g of fish meat sample was placed in a 25 ml colorimetric tube, and 10 ml of acetone was added. The mixture was vortexed for 5 min and centrifuged at 6000 rpm min^−1^ for 10 min. The supernatant was filtered and subjected to GC-MS analysis. The GC-MS system was equipped with an Agilent HP-5MS column (30 m × 0.25 mm × 0.25 μm) and an ISQ™ 7000 single quadrupole mass spectrometer. The injection port temperature was set at 220 °C, and 1 μL of sample was injected in a splitless mode. The column temperature program was as follows: initial temperature at 50 °C (held for 5 min); increased to 100 °C at 3 °C min^-1^ (held for 2 min ); increased to 140 °C at 4 °C min^-1^ (held for 1 min); and then increased to 250 °C at 5 °C min^-1^ (held for 5 min). The SCAN mode was used for scanning within a range of 40–400 m/z. The ion source temperature was set to 240 °C, and the transfer line temperature was 220 °C (Supplementary Table. 1).

### Statistics and reproducibility

Data were presented as mean ± SD (*n* = 3). Student’s t-test was used to determine the significance of the differences between two groups. Fluorescence intensity and gray scale were analyzed using Image J software. *p* < 0.05 was considered statistically significant.

### Reporting summary

Further information on research design is available in the Nature Portfolio Reporting Summary linked to this article.

## Supplementary information

Supplementary Materials

Description of Additional Supplementary Materials

Reporting Summary

## Source data

Source data

## Data Availability

All data generated and/or analyzed during this study are included in this published article and supplementary files. The source data supporting the findings of this study are provided in the Supplementary Data file. Additional information is available from the corresponding author upon reasonable request. Source data are provided with this paper.
