# Supplementary material for: A spontaneously immortalized muscle stem cell line (EfMS) from brown-marbled grouper for cell-cultured fish meat production
Source: Commun Biol. 2024 Dec 24;7:1697. doi: 10.1038/s42003-024-07400-1 (PMC11668886; doi:10.1038/s42003-024-07400-1)

Supplementary Fig. 1

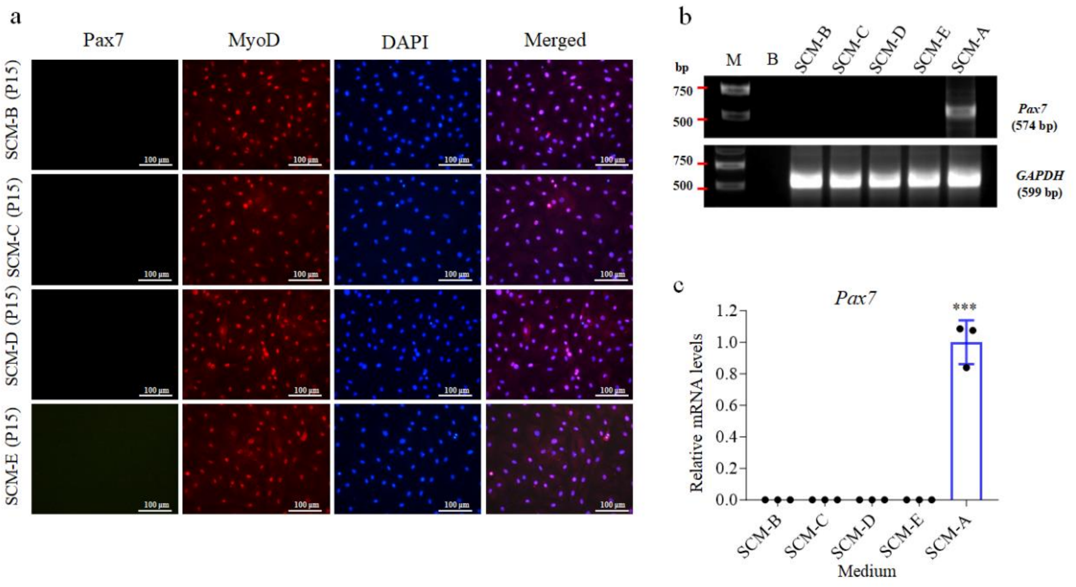

Supplementary Fig. 1 | Stemness maintenance of EfMS cells in different culture media. a, Immunofluorescence staining of Pax7 (green) and MyoD (red) in the EfMS cells at passage 15 and cultured in SCM-B, C, D and E, respectively. Nuclei were labeled by DAPI (blue). Scale bar: 100 μm. **b,** Agarose gel electrophoresis results of semi-quantitative RT-PCR amplification products of *Pax7* and *GAPDH* genes from the EfMS cells at passage 15 and cultured in SCM-B, C, D and E, respectively. M, DNA marker. B, Blank control. **c,** Statistical results of gray values of each band in e, error bars indicate SD, n= 3. Triple asterisks (***) stand for $p < 0.001$.

Supplementary Fig. 2

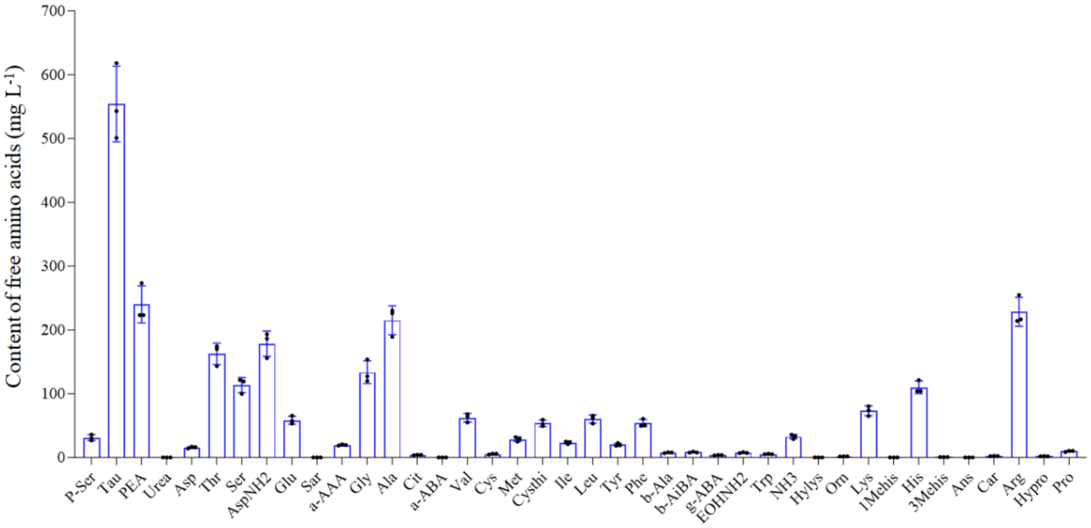

Supplementary Fig. 2 | Analysis of free amino acid composition and content in grouper muscle extract.

Supplementary Fig. 3

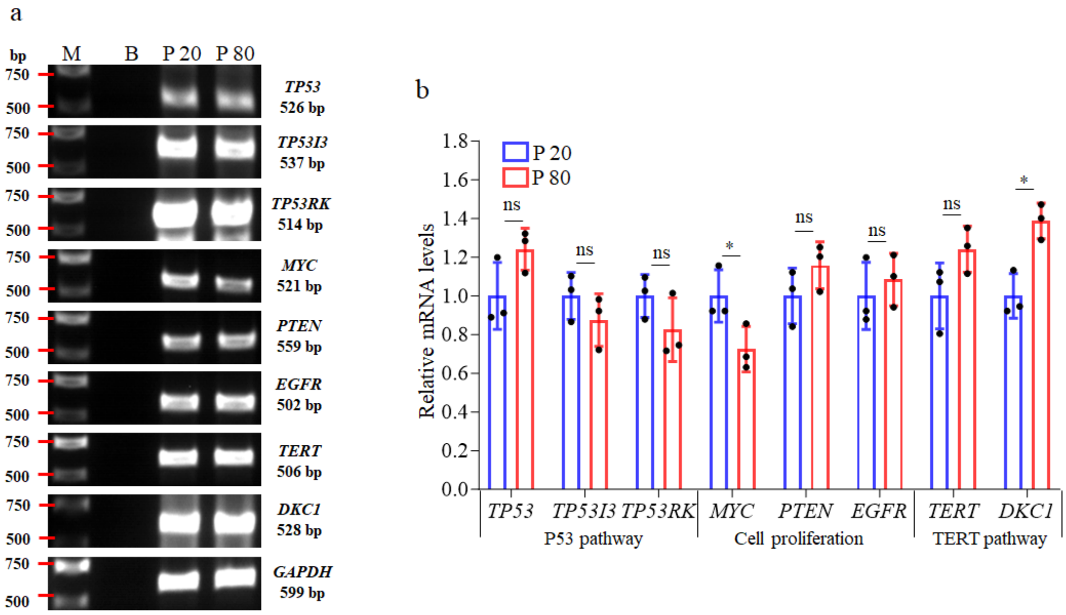

Supplementary Fig. 3 | Examination of the impact of the spontaneous immortalization event on the genetic stability of the EfMS cell line. a, Agarose gel electrophoresis results of semi-quantitative RT-PCR amplification of *TP53*, *TP53I3*, *TP53RK*, *MYC*, *PTEN*, *EGFR*, *TERE*, *DKC1* and *GAPDH* from the EfMS cells at passage 20 and 80, respectively. **b,** Statistical results of gray values of each band in a, error bars indicate SD, n= 3. M, DNA marker; B, blank control. Asterisk (*) stands for $p < 0.05$. ns stands for $p > 0.05$.

Supplementary Fig. 4

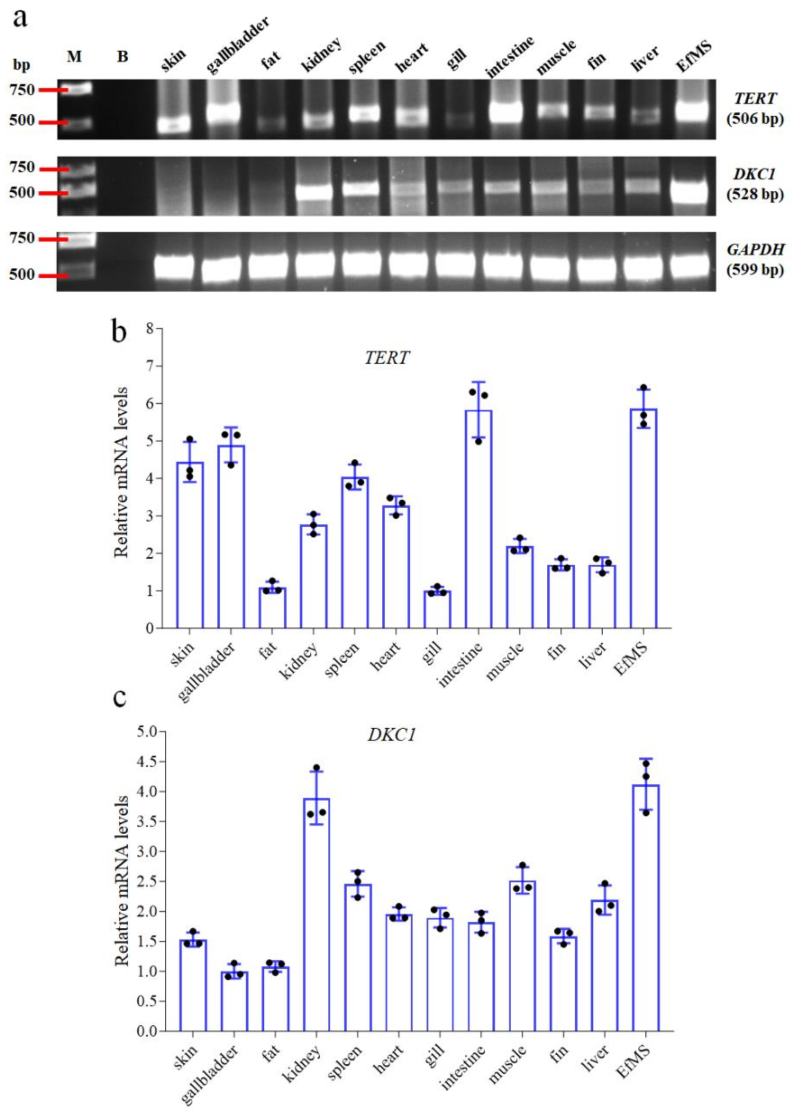

Supplementary Fig. 4 | Expression levels of *TERT* and *DKC1* in EfMS cells and different tissues of brown-marbled grouper. a, Agarose gel electrophoresis results of semi-quantitative RT-PCR amplification of *TERE, DKC1* and *GAPDH* from skin, gallbladder, fat, kidney, spleen, heart, gill, intestine, muscle, fin, liver and EfMS cells, respectively. **b,** Statistical results of gray values of each band in a, error bars indicate SD, n= 3. M, DNA marker; B, blank control.

Supplementary Table 1

No	Volatile Compounds	Concentrations (μg kg^{-1})	
		NM	CCM
Aldehydes (3)			
1	Isobutyraldehyde	2.09 ± 0.12	ND
2	Isovaleraldehyde	ND	1.34 ± 0.02
3	2-Methylhexadecanal	ND	17.23 ± 3.24
Ketones (3)			
4	4-Hydroxy-4-methyl-2-pentanone	58.02 ± 7.56	67.35 ± 11.54
5	2-Nonadecanone	3.01 ± 0.07	ND
6	Mesityl oxide	ND	3.12 ± 0.09
Alcohols (8)			
7	Acetol	11.33 ± 2.86	2.61 ± 0.28
8	Geranylgeraniol	1.25 ± 0.02	ND
9	4-Methoxy-1-butanol	ND	2.08 ± 0.04
10	1-Pentadecanol	ND	2.34 ± 0.07
11	1-Heptadecanol	ND	0.74 ± 0.01
12	1-Nonadecanol	ND	1.30 ± 0.11
13	Phytol	ND	1.43 ± 0.04
14	Cholesterol	ND	34.15 ± 8.53
Acids (2)			
15	Acetic acid	35.40 ± 4.61	ND
16	Propionic acid	3.01 ± 0.57	6.66 ± 0.28
Hydrocarbons (4)			
17	n-Eicosane	ND	26.72 ± 4.65
18	2,6,10,14-Tetramethylpentadecane	ND	14.96 ± 2.87
19	cis-9-Tricosene	ND	1.22 ± 0.09
20	Squalene	ND	31.80 ± 2.58
Esters (3)			
21	cis-6-Octadecenoic acid methyl ester	ND	1.19 ± 0.05
22	2-Monopalmitin	ND	3.60 ± 0.41
23	1,2-Dipalmitoylglycerol	ND	3.40 ± 0.08
Others (8)			
24	N-Formylglycine	11.59 ± 2.53	ND
25	N,N-Dimethylethylamine	8.0 ± 1.22	ND
26	2-Isobutyl oxirane	0.63 ± 0.03	ND
27	2-Hydroperoxyheptane	1.42 ± 0.16	3.61 ± 0.65

28	tert-Amylamine	1.47 ± 0.25	ND
29	Palmitoyl chloride	1.11 ± 0.09	2.34 ± 0.14
30	4-Trimethyl-3-cyclohexene-1-methylamine	8.60 ± 0.16	ND
31	(2-Amino-2-methylpropyl)(propan-2-yl)amine	ND	4.01 ± 0.84

Notes: NM: Natural fish meat. CCM: Cell-cultured fish meat. ND: Not Detected.

Raw Gel Images

Fig.1e-CO1

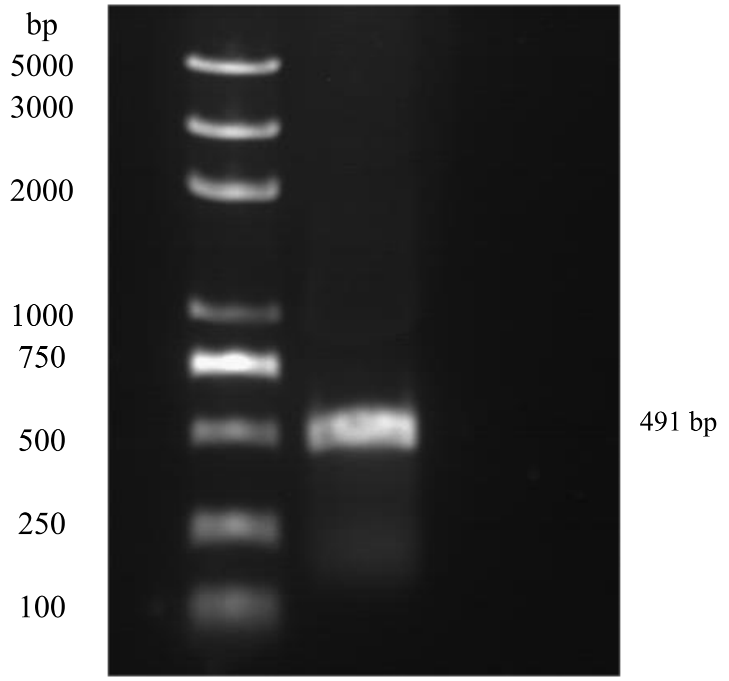

Fig.2b-PAX7

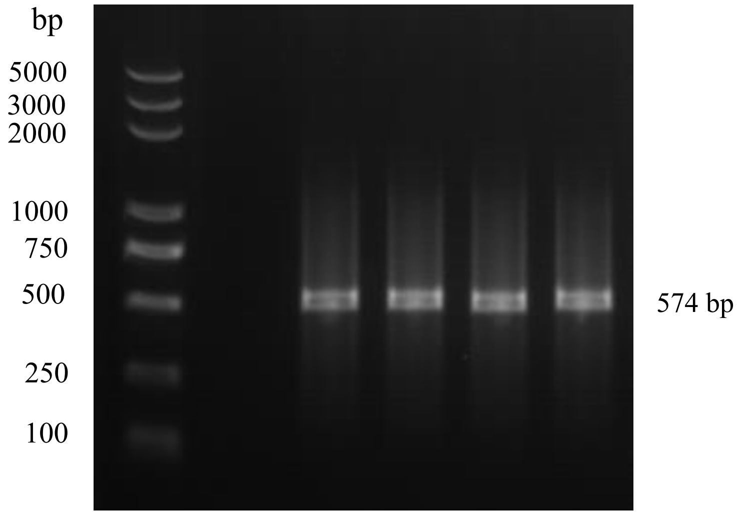

Fig.2b-GAPDH

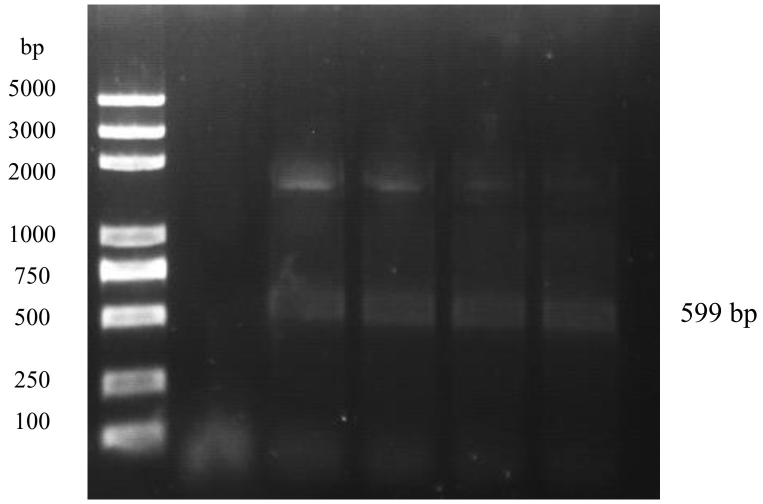

Fig.2e-PAX7

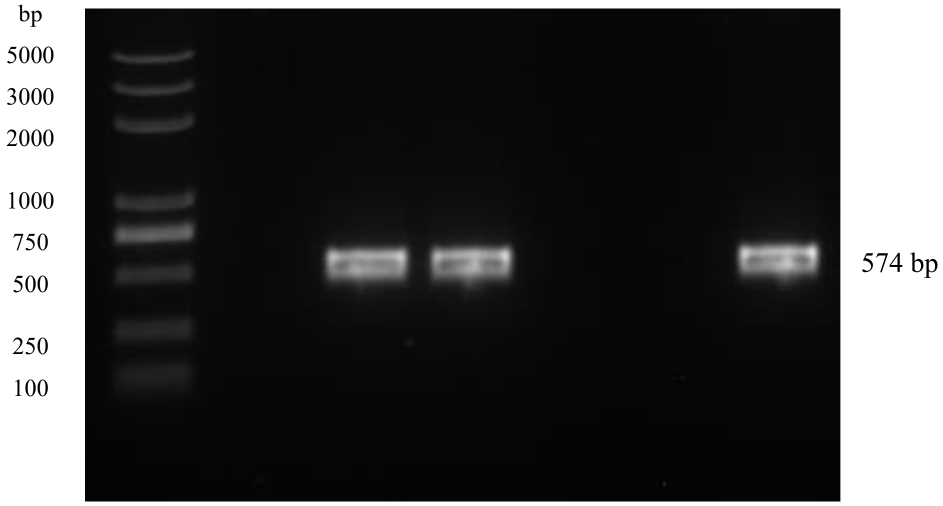

Fig.2e-GAPDH

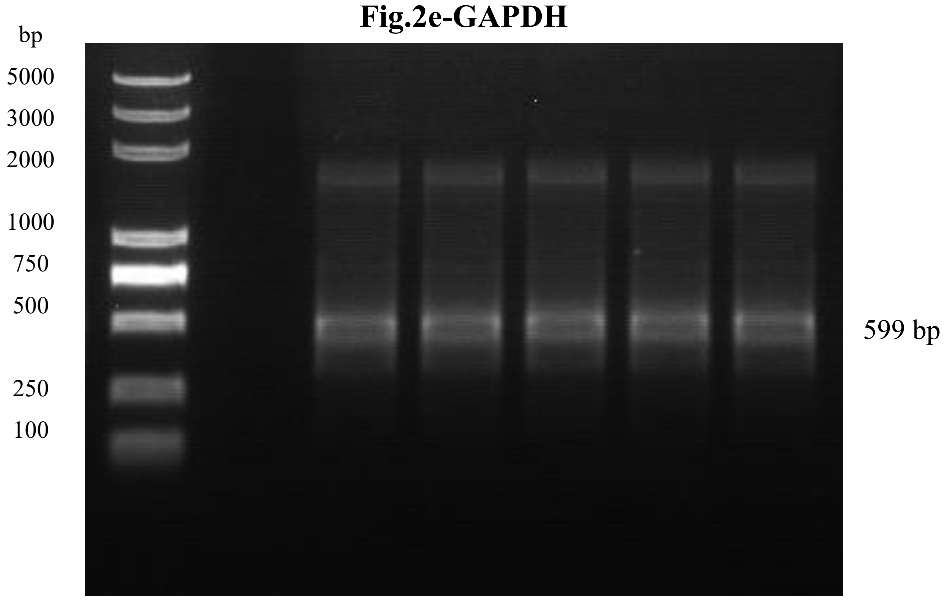

Fig.3i-ACTB

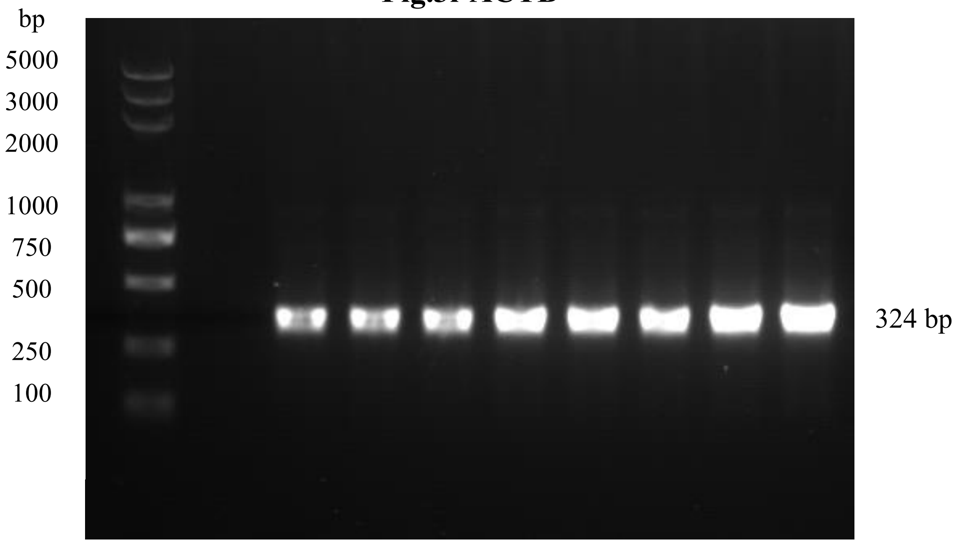

Fig.3i-GAPDH

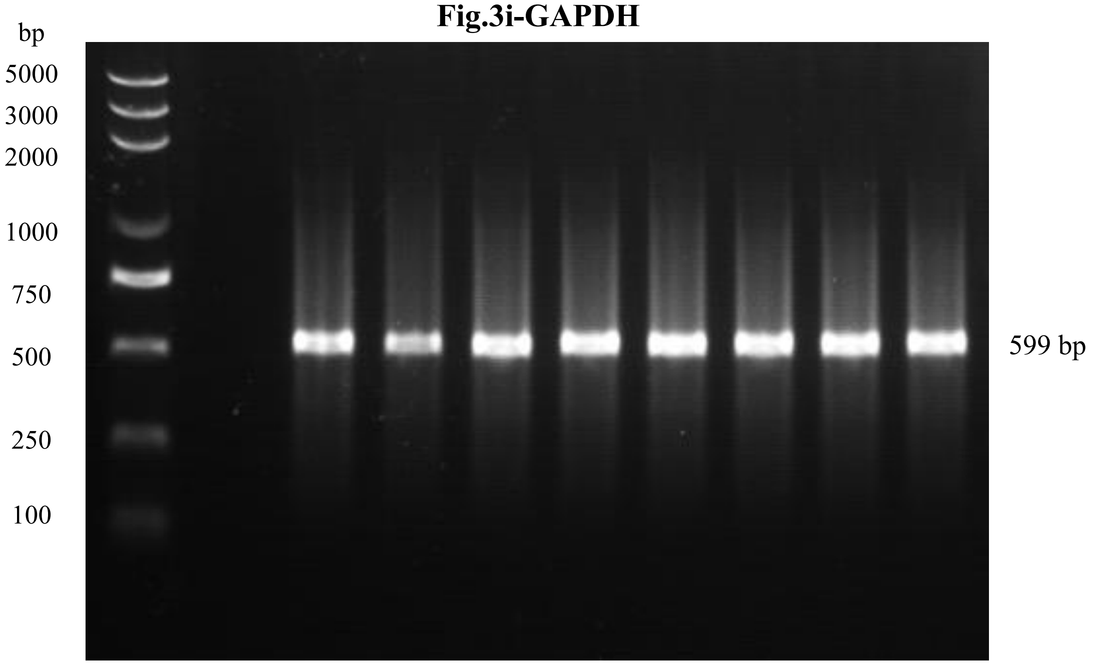

Fig.3k-MHC

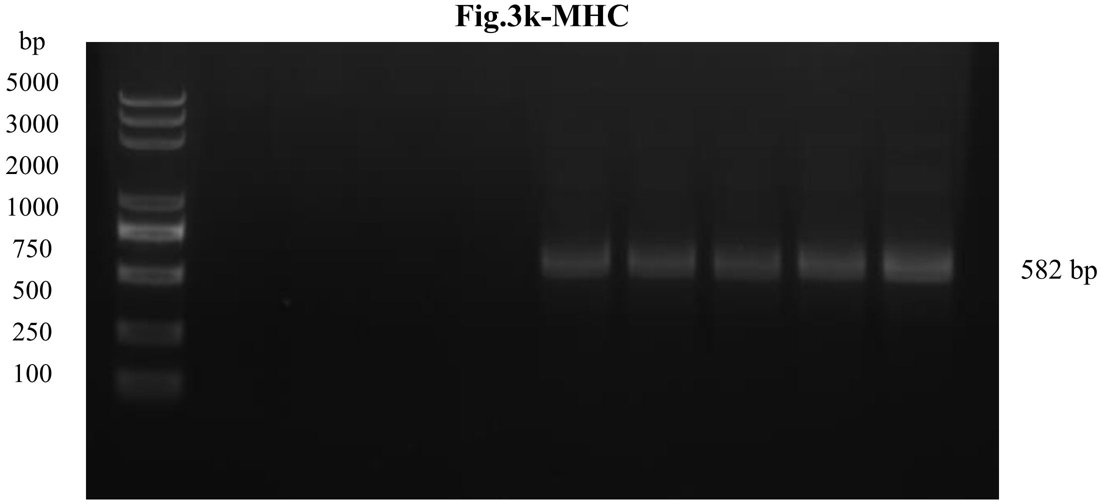

Fig.3k-GAPDH

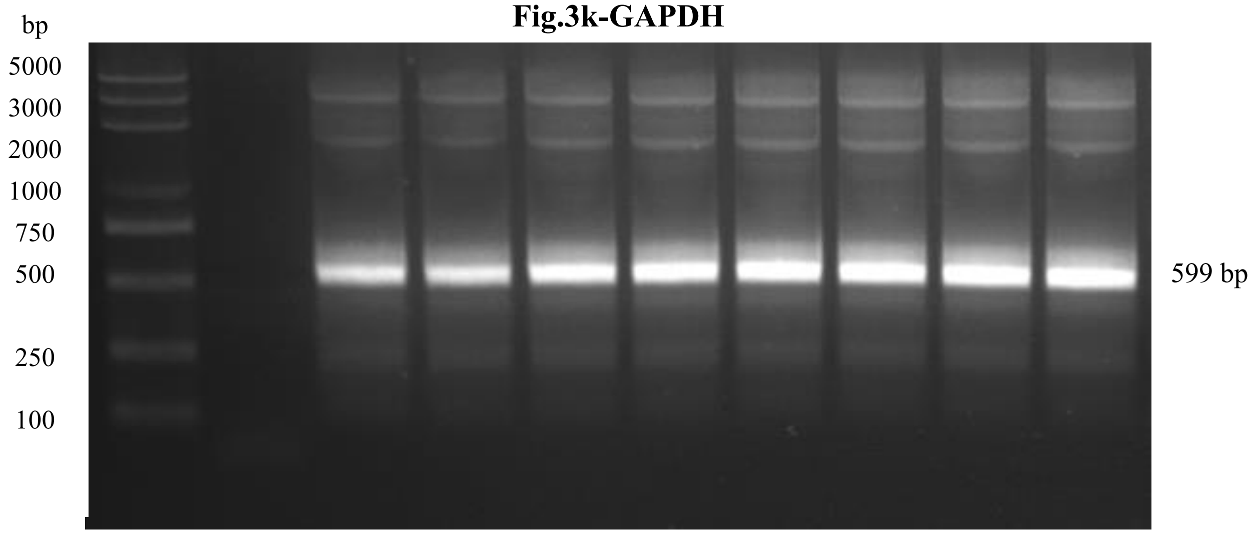

Fig.3m-Myogenin

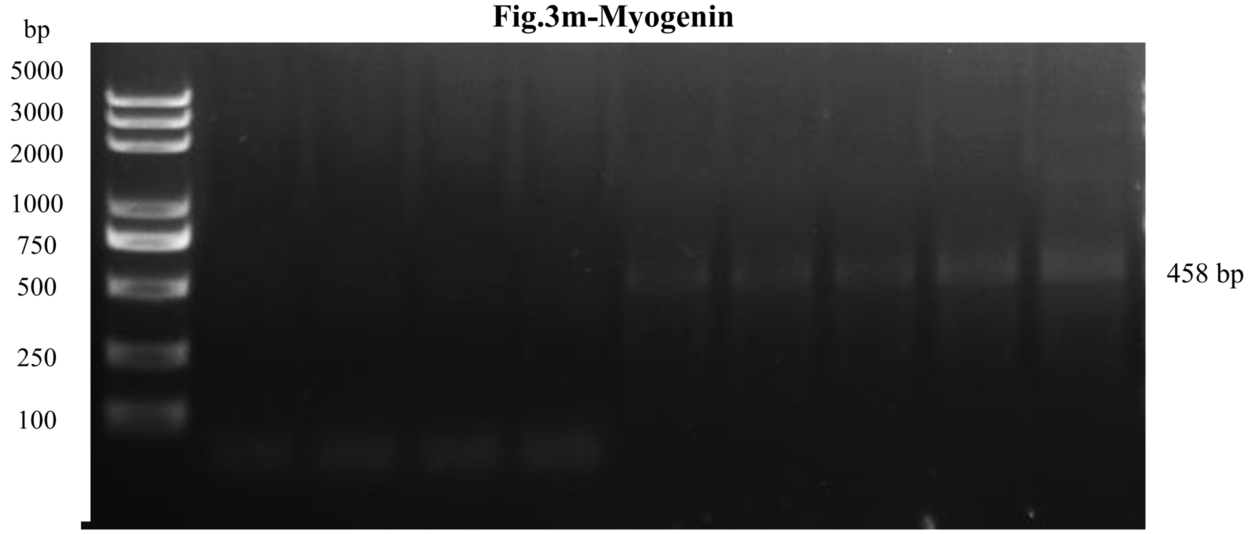

Fig.3m-GAPDH

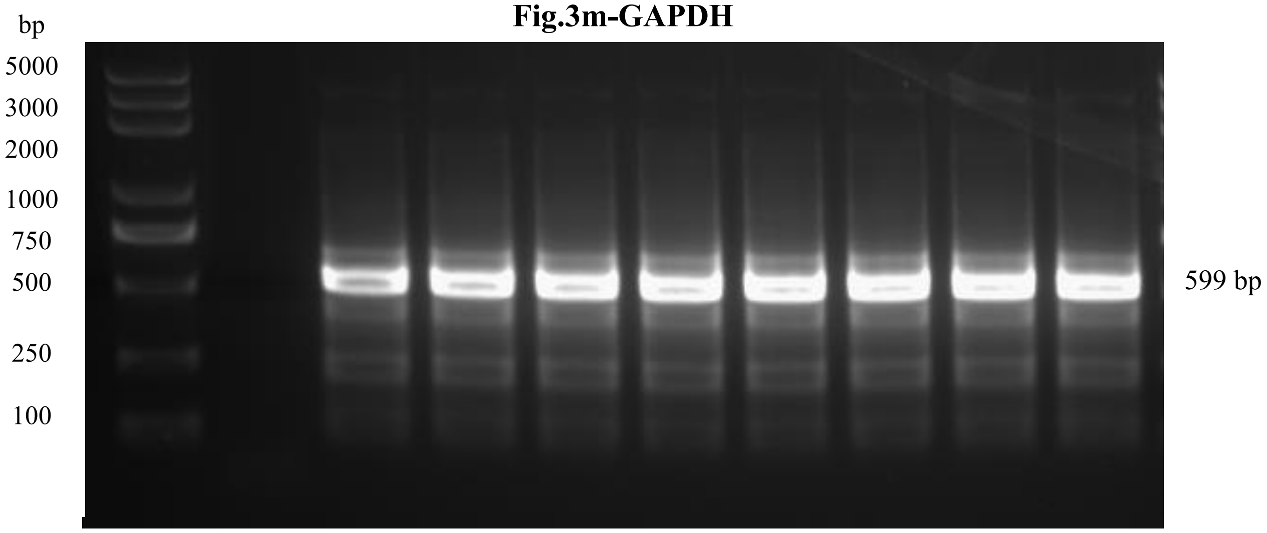

Fig.5e-ACTB

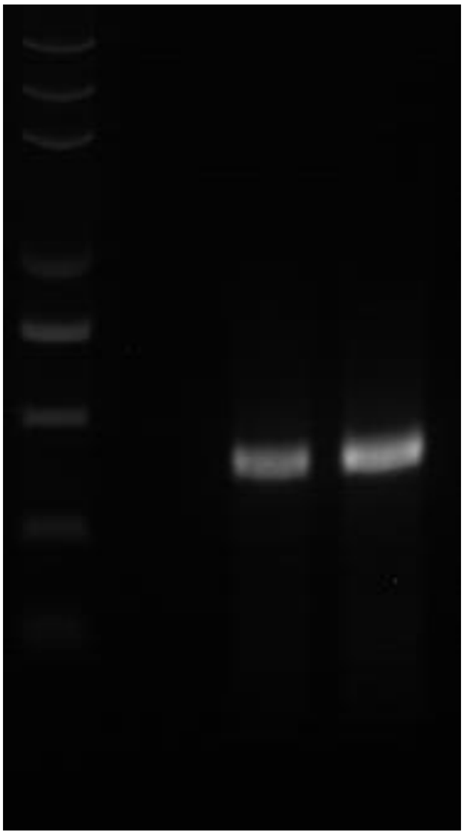

Fig.5e -Myogenin

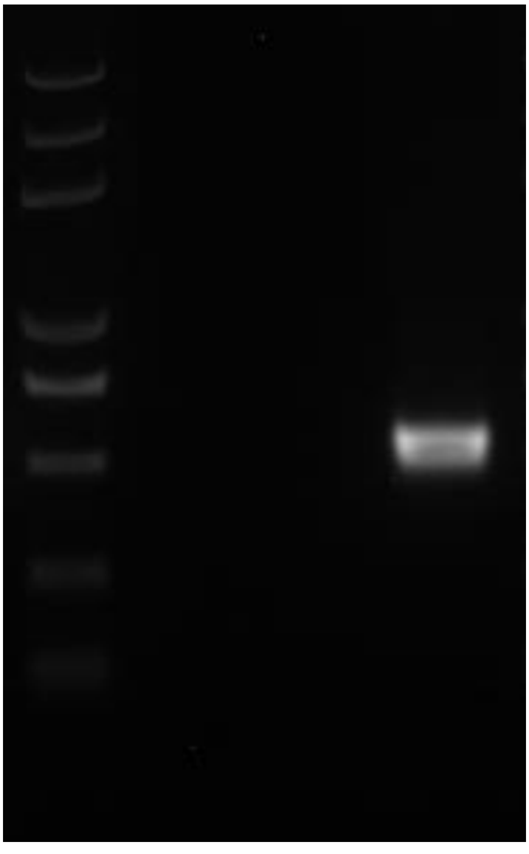

Fig.5e-MHC

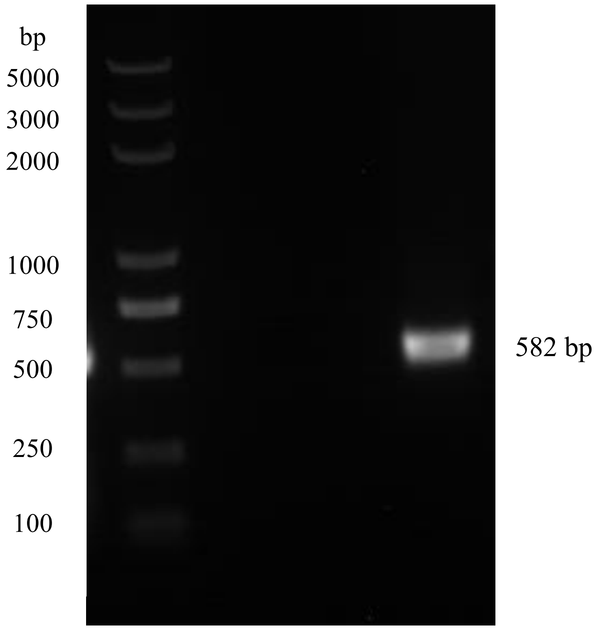

Fig.5e-PAX7

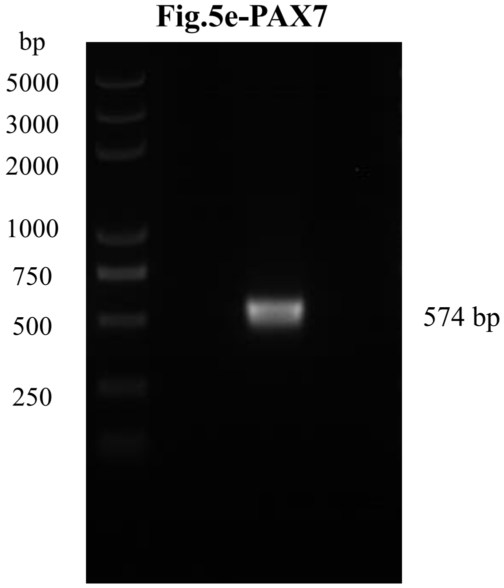

Fig.5e-MyoD

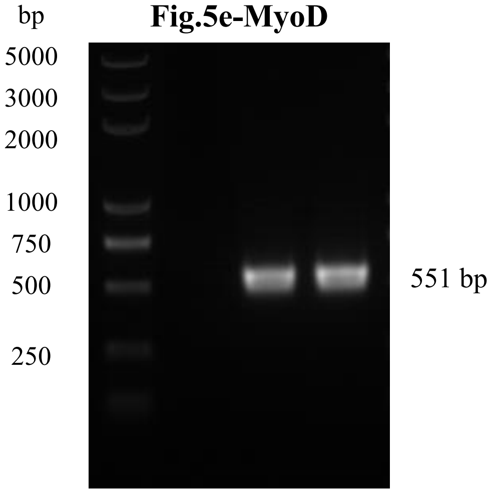

Fig.5e-GAPDH

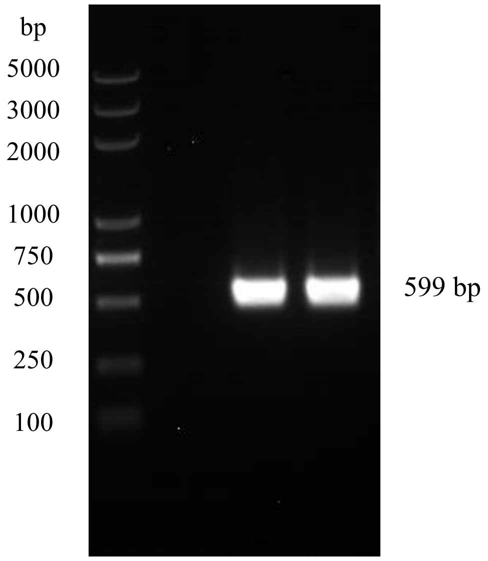

Fig.6b-LPL

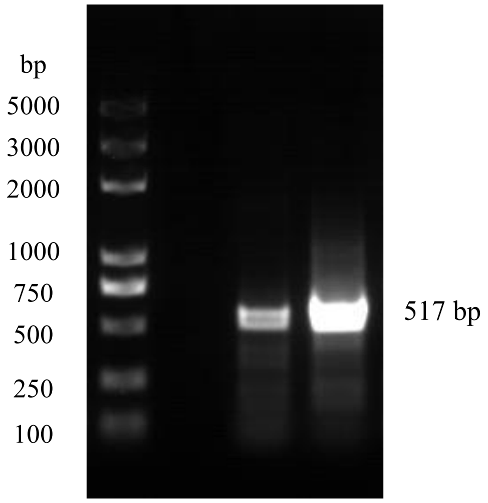

Fig.6b-PPARγ

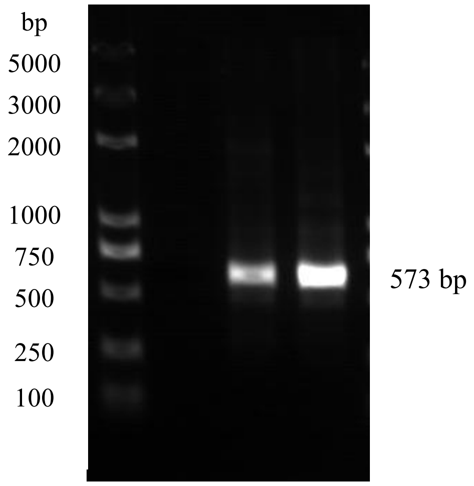

Fig.6b-Leptin

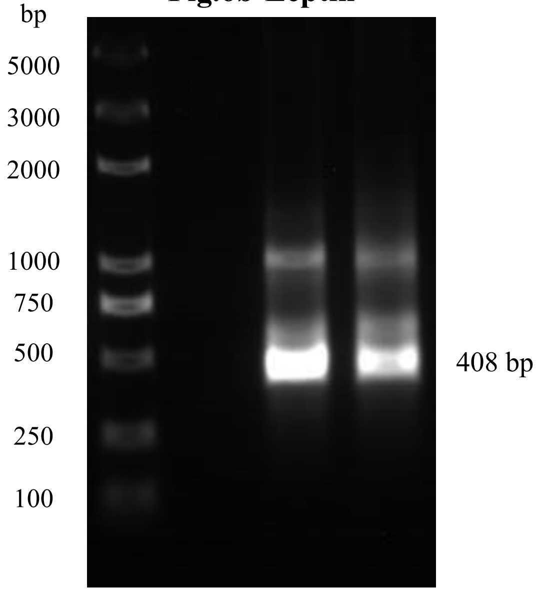

Fig.6b-C/EBPα

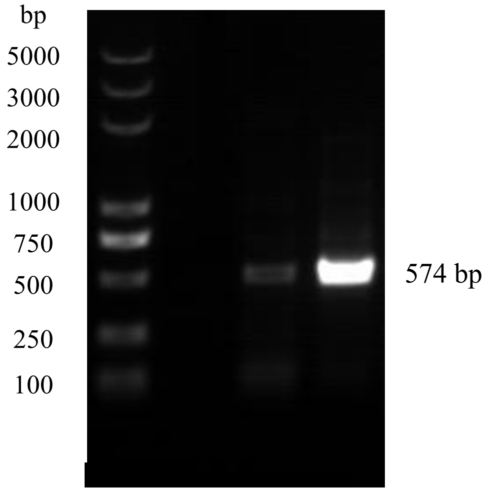

Fig.6b-CD73

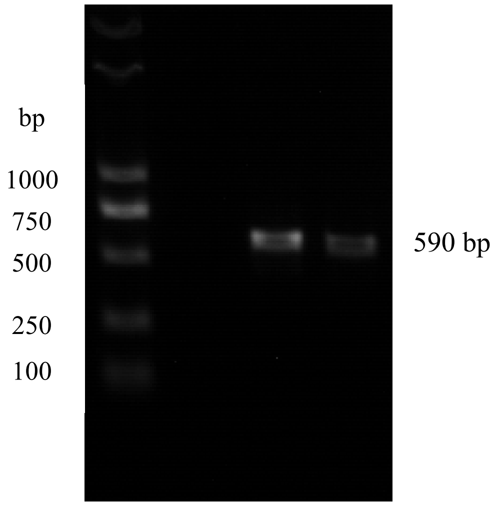

Fig.6b-CD105

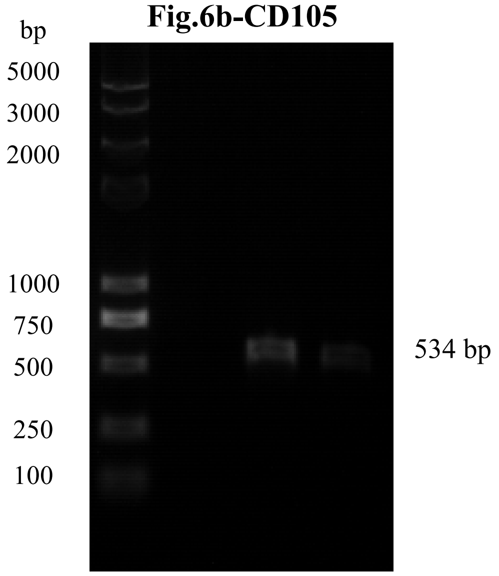

Fig.6b-GAPDH

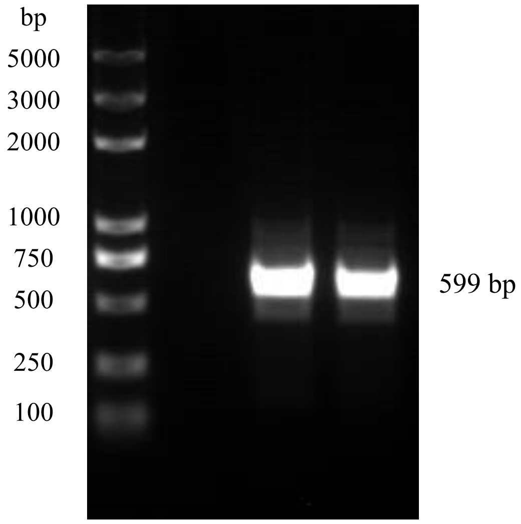

Supplementary Fig.1b-PAX7

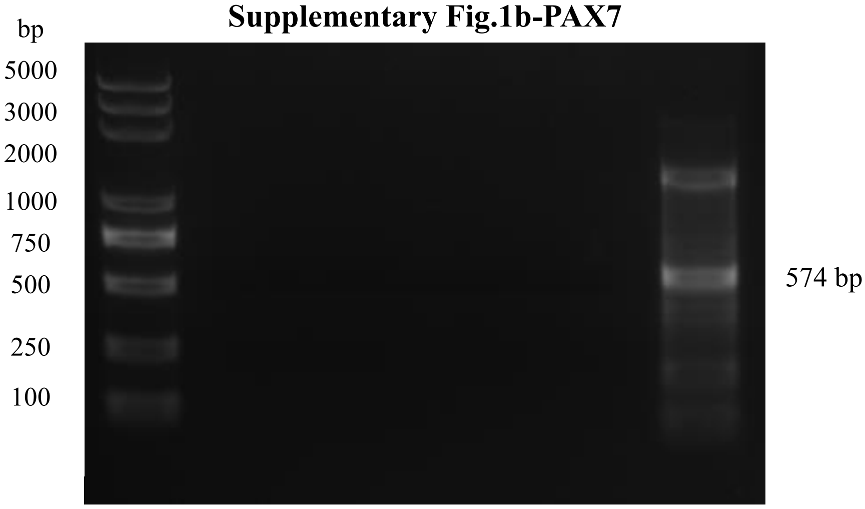

Supplementary Fig.1b-GAPDH

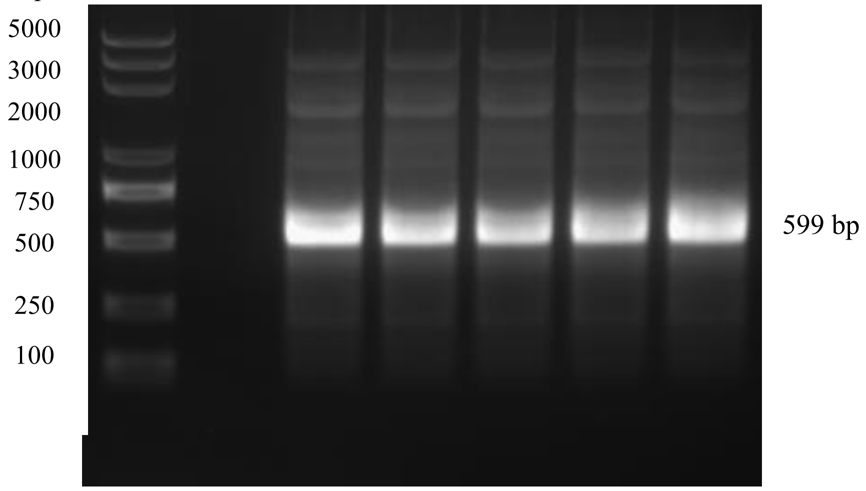

Supplementary Fig.3a-TP53

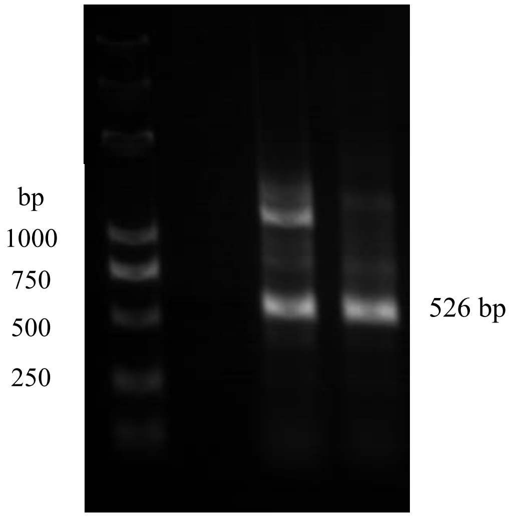

Supplementary Fig.3a-TP53I3

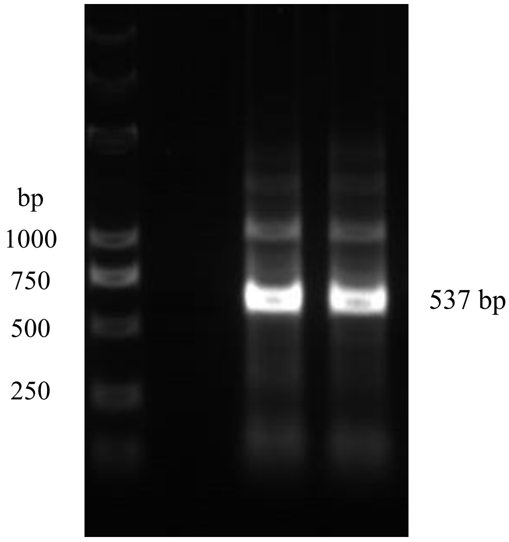

Supplementary Fig.3a-TP53RK

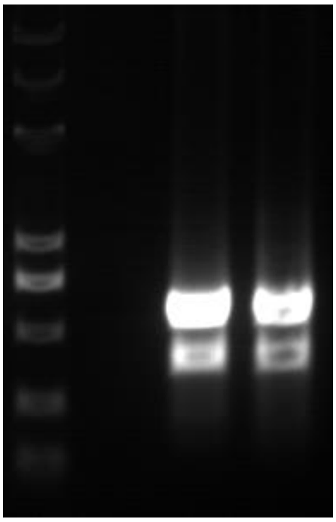

Supplementary Fig.3a-PTEN

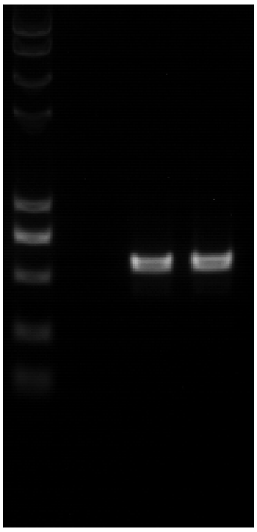

Supplementary Fig.3a-MYC

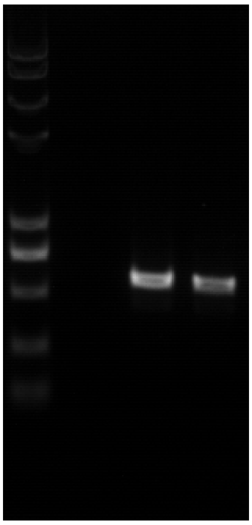

Supplementary Fig.3a-EGFR

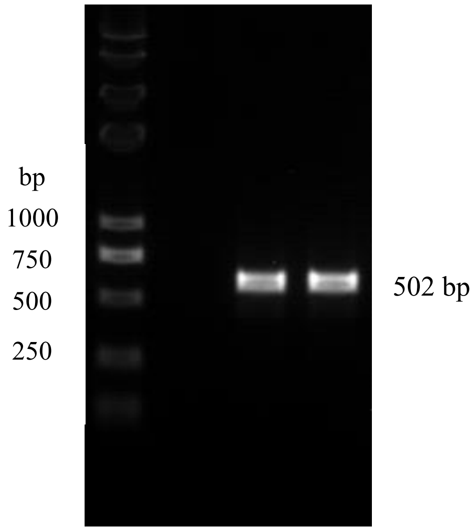

Supplementary Fig.3a-TERT

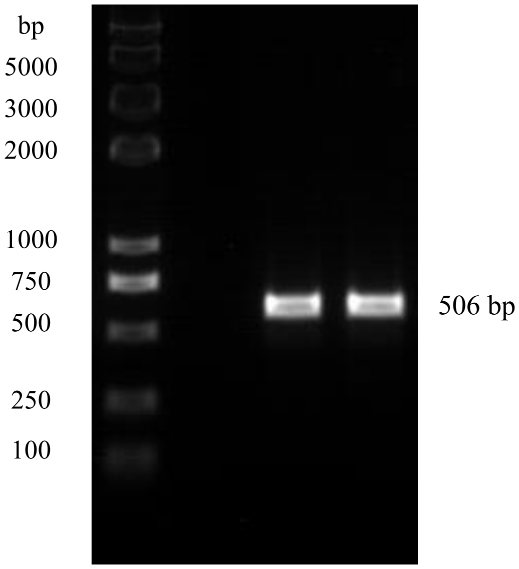

Supplementary Fig.3a-DKC1

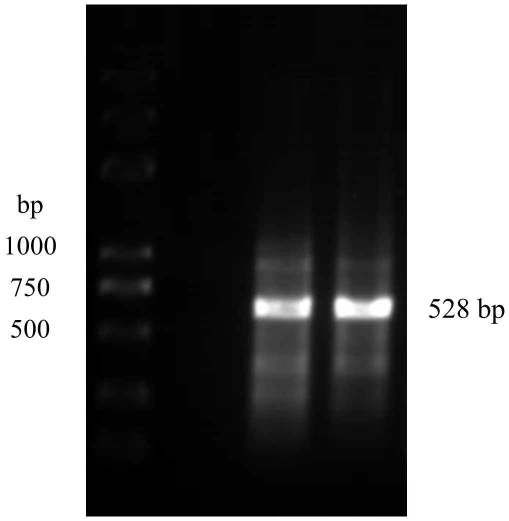

S[illegible]plementary Fig.3a-GAPDH

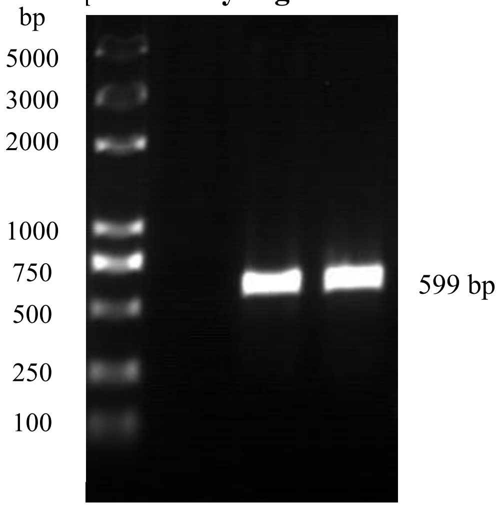

Supplementary Fig.4a-TERT

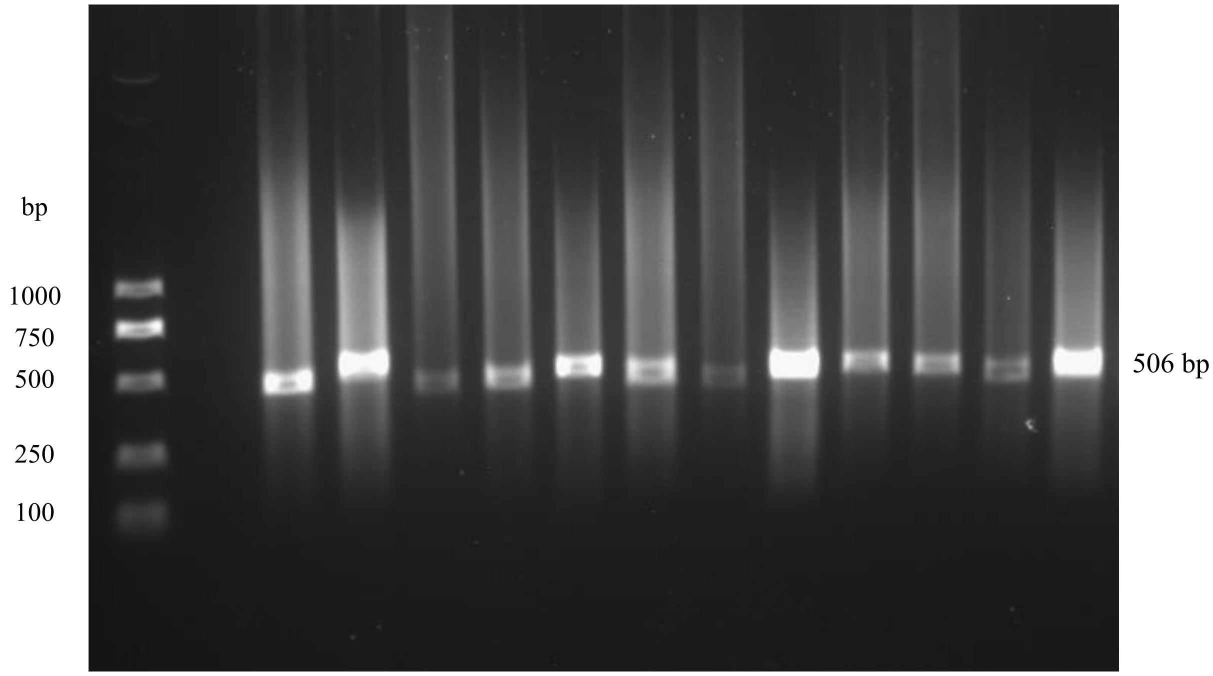

Supplementary Fig.4a-DKC1

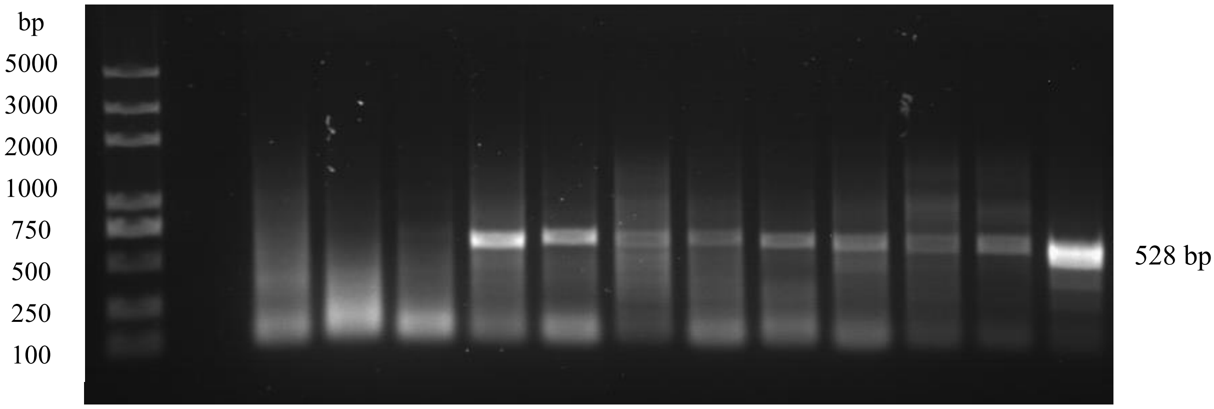

Supplementary Fig.4a-GAPDH

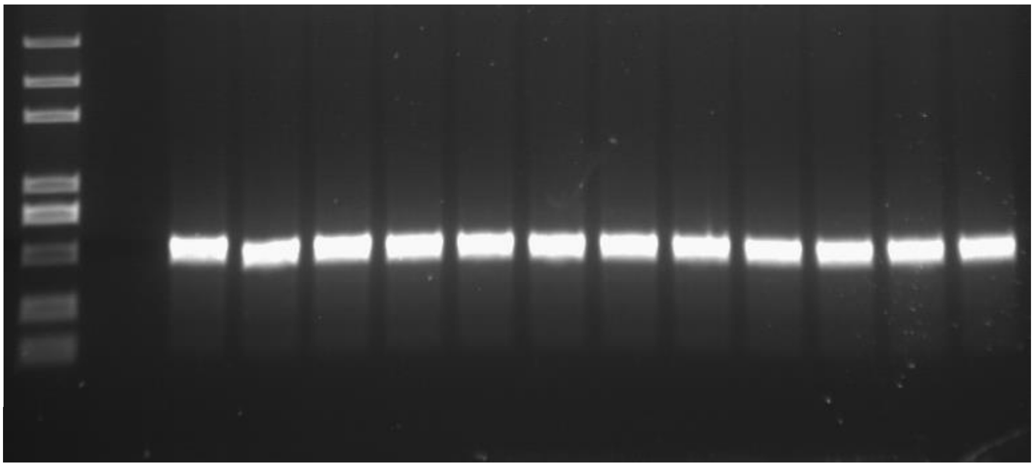

Supplement: Supplementary file 1 — Supplementary Materials [file 42003_2024_7400_MOESM1_ESM.pdf]
